# A 3D‐Printed SFE‐Graded Alloy Mitigates Cryogenic Discontinuous Plastic Flow and Hydrogen Embrittlement

**DOI:** 10.1002/advs.77732

**Published:** 2026-09-11

**Authors:** Renhao Wu, Ao Geng, Peihao Geng, Hyojin Park, Eun Seong Kim, Sang‐Ho Oh, Stephan Schönecker, Xiaoqing Li, Tianlong Zhang, Shuhui Li, Byeong‐Joo Lee, Hyoung Seop Kim

**Affiliations:** ^1^ Shanghai Key Laboratory of Digital Manufacture for Thin‐Walled Structures School of Mechanical Engineering Shanghai Jiao Tong University Shanghai China; ^2^ Advanced Institute for Materials Research (WPI‐AIMR) Tohoku University Sendai Japan; ^3^ Graduate Institute of Ferrous & Eco Materials Technology Pohang University of Science & Technology Pohang South Korea; ^4^ Department of Materials Science & Engineering Pohang University of Science & Technology Pohang South Korea; ^5^ Department of Materials Science and Engineering Applied Materials Physics KTH – Royal Institute of Technology Stockholm Sweden; ^6^ Department of Mechanical and Aerospace Engineering The Hong Kong University of Science and Technology Kowloon China

**Keywords:** gradient nanotwin, hydrogen embrittlement resistance, in situ additively manufactured graded (CoCrMn)_100‐2x_Fe_x_Ni_x_, suppressed discontinuous plastic flow, synergistic cryogenic strength‐ductility

## Abstract

The lunar polar permanently shadowed regions, rich in water ice for hydrogen production, impose a trade‐off in FCC alloys: lowering stacking‐fault energy (SFE) enhances hydrogen embrittlement resistance but exacerbates cryogenic discontinuous plastic flow (DPF). To address this, we designed and fabricated via additive manufacturing a compositionally graded (CoCrMn)_100__−__2__x_Fe_x_Ni_x_ alloy with a single‐phase FCC structure. Guided by thermodynamic calculations and first‐principles predictions, the gradient‐structured build exhibits spatial SFE and microstructural gradients. Quasi‐static tensile tests from 298 K down to 20 K reveal significant strengthening with retained high ductility, while DPF is strongly suppressed, showing only minor stress drops. Mechanistically, the graded architecture partitions deformation: high‐SFE regions promote dislocation glide and cellular substructures, whereas low‐SFE regions enable planar slip and deformation nanotwinning, delocalizing strain and suppressing avalanche‐like dislocation activity. Under hydrogen charging at 298 K, embrittlement resistance is preserved via a dual mechanism combining deformation‐induced nanotwins and a Mn concentration gradient. This synergy balances dislocation mobility and hydrogen trapping, offering a new paradigm for simultaneous improvement of cryoplasticity and hydrogen compatibility. Our work establishes a multidisciplinary design strategy for alloys targeting extreme hydrogen and cryogenic environments.

## Graphical abstract

A novel strategy features gradient composition and stacking fault energy‐tuned (CoCrMn)_100‐2x_Fe_x_Ni_x_ fabricated via in situ alloying additive manufacturing. This design circumvents cryogenic discontinuous plastic flow and hydrogen embrittlement of metallic materials under quasi‐static loading and hydrogen‐rich environment relevant to lunar polar explorations and resource utilization, and the inherent dilemma of stacking fault suppression.

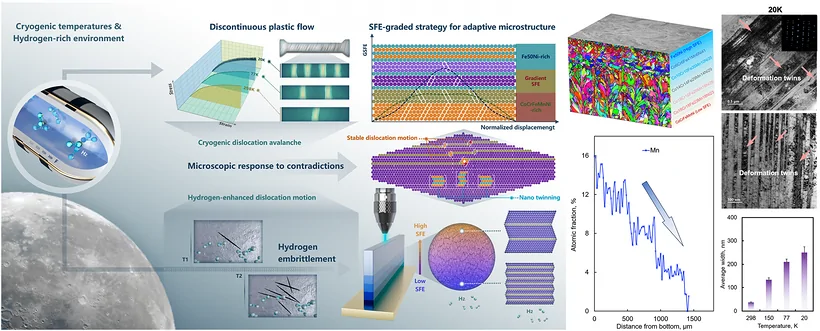

## 1 Introduction

As deep space exploration advances, the polar regions of the Moon have attracted growing interest as strategic sites for future outposts, owing to their abundant water ice resources that can be electrolyzed to generate hydrogen for propulsion and life support systems. Nevertheless, the essential metallic components used for hydrogen containment and conveyance are confronted with a fundamental challenge: they are required to endure both quasi‐static mechanical loading under cryogenic conditions and exposure to hydrogen‐rich environments [1, 2, 3, 4]. Under these coupled extreme conditions, conventional structural materials are susceptible to severe instabilities, including discontinuous plastic flow (DPF) [5, 6], characterized by jerky flow and unstable, oscillatory stress–strain behavior due to the intermittent pinning and unpinning of dislocations, and hydrogen embrittlement (HE) [7, 8], a hydrogen‐assisted failure process that drastically reduces fracture toughness and promotes subcritical crack growth. Such degradation seriously compromises the structural integrity and mission safety. A synergistic combination of outstanding cryogenic mechanical performance, resistance to DPF, and HE immunity in structural materials, especially the commonly used face‐centered cubic (FCC) metals, remains a major unresolved challenge.

The potential DPF observed in FCC alloys with low SFE at cryogenic temperatures is primarily described as dislocation avalanches at low temperatures. Numerous studies have revealed that a close correlation exists between SFE and the mechanical responses of metallic materials [9, 10, 11, 12, 13]. In practice, the SFE of FCC materials exhibits high sensitivity to environmental variables, including principal and trace alloying elements as well as temperature, through intricate coupling effects [14, 15]. Increased SFE generally facilitates cross‐slip of dislocations, thereby mitigating the risk of failure caused by excessive dislocation pile‐ups and ensuring sustained work hardening. Moreover, a low SFE promotes planar slip, localizing dislocation glide and facilitating the intense interactions that lead to the formation of potent pinning points [16]. Meanwhile, hydrogen has been found to reduce the SFE of the matrix and promote nanotwining in FCC systems [17, 18], impeding the rapid accumulation of hydrogen‐induced dislocations at crack tips [19, 20]. Therefore, the ability to tune mechanical performance through SFE engineering has motivated the pursuit of compositionally graded or functionally layered materials with tailored deformation modes and improved resistance to plastic instability.

Despite these advances, the reported strategies have addressed cryogenic DPF and HE resistance in isolation rather than as coupled design criteria. In particular, SFE engineering, compositional grading, nanotwin‐induced HE resistance [9, 10, 11, 12, 13], and additive manufacturing of HEAs [19, 20, 21, 22] have each been individually explored, yet no prior study has demonstrated an integrated approach that simultaneously resolves the HE‐DPF trade‐off within a single alloy system. Additionally, graded architectures often suffer from discontinuous property transitions and interfacial incompatibility, though these issues are less detrimental in homogeneous FCC alloys [23, 24]. Consequently, a systematic strategy that synergistically combines continuous SFE tailoring, cryogenic DPF suppression, and HE mitigation remains conspicuously absent.

**FIGURE 1:**
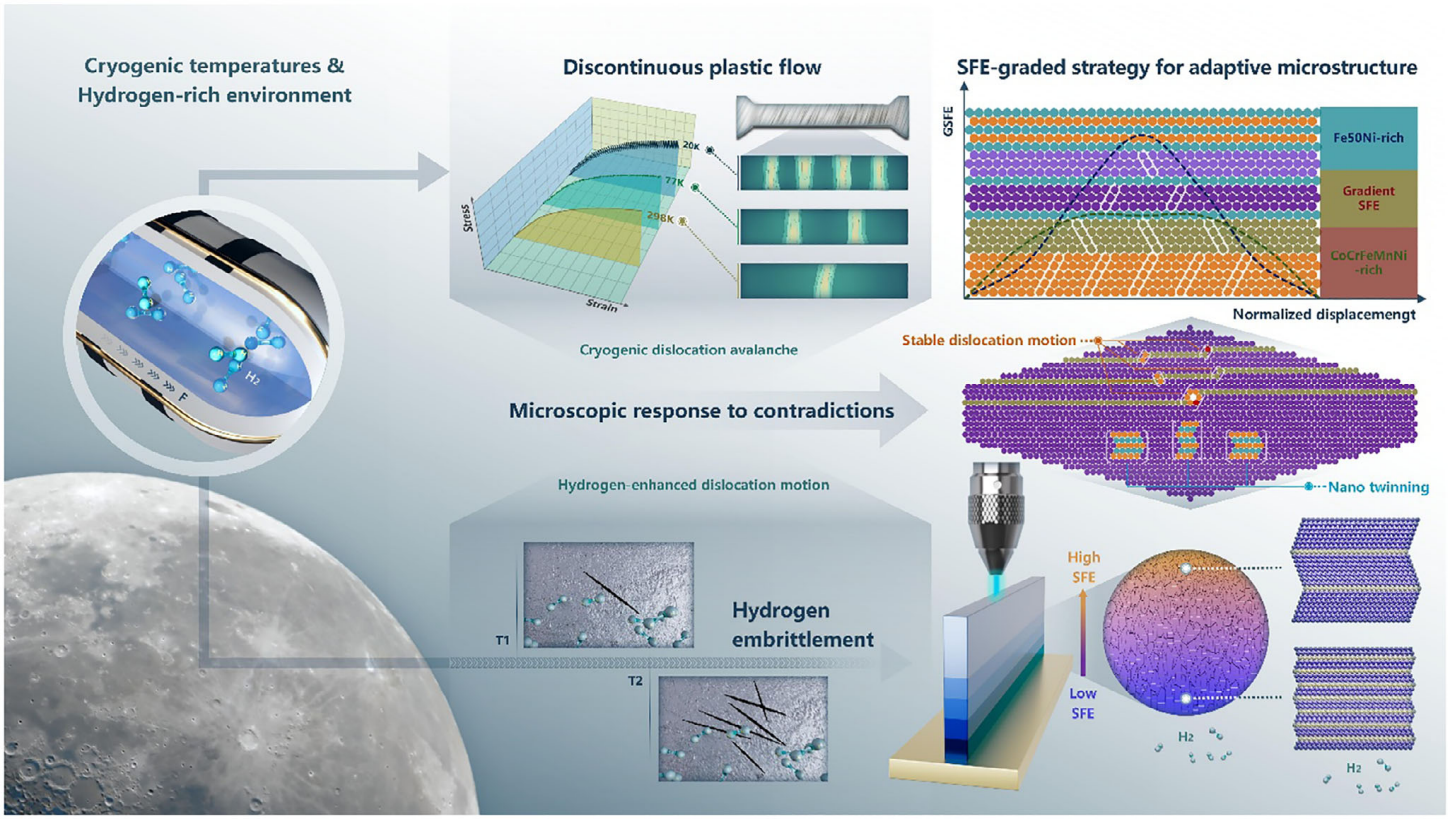
Design concept addressing DPF and HE of metallic materials under quasi‐static loading in cryogenic to ambient temperatures and a hydrogen‐rich environment relevant to lunar polar explorations and resource utilization, and the inherent dilemma of stacking fault suppression via microstructural design. A novel strategy features gradient composition and SFE‐tuned (CoCrMn)_100‐2x_Fe_x_Ni_x_ fabricated via in situ alloying additive manufacturing (right).

Here, we propose that a gradient SFE design offers a pathway to resolve this paradox: by locally increasing SFE to suppress cryogenic DPF while reducing SFE to mitigate HE, a spatially tuned microstructure can be achieved, as schematically illustrated in Figure 1. This represents a paradigm‐shifting material‐design strategy that effectively implements the principle of “applying the right material at the right location” [25]. Guided by this gradient‐design approach to surpass the limits of uniform composition, we have fabricated a (CoCrMn)_100‐2x_Fe_x_Ni_x_ alloy with a continuous composition gradient. This material exhibits a spatially varying chemical composition and SFE, ranging from the CoCrFeMnNi‐rich region to the Fe_50_Ni‐rich region, enabling the programmed formation of a functionally graded microstructure at the microscale. We demonstrate that this heterogeneity manipulates deformation mechanisms: in CoCrFeMnNi‐rich with low‐SFE regions, deformation preferentially activates nanoscale twinning; whereas in high‐SFE regions, stable dislocation glide dominates. This work establishes a novel paradigm for designing integrated structural‐functional materials capable of withstanding extreme environments through the integration of multiscale modeling and additive manufacturing strategy.

## 2 Experimental Strategy and Methods

### 2.1 Materials and Sample Preparation

In this study, an alloy with a single FCC phase was fabricated via laser‐directed energy deposition (DED, MX‐lab, InssTek Co., Korea), guided by phase diagram calculations. This method enables precise compositional control between Fe_50_Ni and equiatomic CoCrFeMnNi. The study aims to unveil the complex coupled gradient effects, facilitating a shift from tunable properties to reliable and predictable performance. The flexibility of DED in constructing multi‐layer architecture is highlighted, establishing its potential as a high‐throughput platform for graded metallic materials. CoCrFeMnNi and Fe_50_Ni powders with an initial particle size ranging from 50 to 100 µm were used in the DED process. A multi‐track laser scanning strategy with a 90° rotation between successive layers was adopted during printing to reduce anisotropy. The process utilized a laser beam diameter of 90 µm with protection gas as pure Ar. Key processing parameters included a laser power of 240 W for CoCrFeMnNi and 220 W for Fe_50_Ni (adjusted based on the powder density to fabricate gradient layers), a scanning speed of 16.7 mm/s, and a hatch spacing of 300 µm. The substrate is stainless steel 316L alloy without pre‐heating. The detailed fabrication procedures and layer composition of the gradient (CoCrMn)_100‐2x_Fe_x_Ni_x_ sample are illustrated in Figures S1 and S2, providing variations of powder feeding rate and mixing ratio with 8 layers.

Prior to mechanical testing, electrochemical hydrogen charging was conducted under cathodic conditions to introduce hydrogen into the matrix. Dog‐bone‐shaped specimens were mechanically ground to a 2000‐grit finish, ultrasonically cleaned in acetone, and rinsed with deionized water. The charging procedure was performed galvanostatically at a constant current density of 100 A·m^−^^2^ for 12 h (charging time, *t_c_*) in 1 L of an aqueous electrolyte composed of NaCl and NH_4_SCN in a 10:1 weight ratio, maintained at 298 K, using an Agilent 6035A potentiostat/galvanostat. Non‐charged control specimens were prepared following identical surface finishing and handling protocols.

To quantify the hydrogen content and compare the trapping and release behavior, thermal desorption analysis (TDA) was utilized. TDA measurements were carried out under a flowing argon atmosphere, with specimens heated from 298 to 650 K at a constant heating rate of 10 K·min^−^^1^. The total hydrogen content was derived by integrating the desorption signal following baseline correction and calibration.

### 2.2 Mechanical Tests and Microstructural Characterizations

Dog‐bone‐shaped samples with nominal gauge dimensions of 6.4 (*x*) × 2.5 (*y*) × 1.2 (*z*) mm^3^ were prepared using electrical discharge machining for uniaxial tensile testing. The tensile direction was aligned perpendicular to the building direction (BD) of the DED samples. Tensile tests were performed using an in‐house‐built testing system equipped with a liquid helium cooling unit for precise temperature control. The slow strain rate tensile test was conducted at 1 × 10^−^^4^ s^−^^1^. For each testing condition, 3 parallel measurements were performed to ensure intra‐batch data reliability. For hydrogen‐related tensile tests, dog‐bone‐shaped samples were first taken for electrochemically hydrogen charged with a 1 L solution of NaCl and NH_4_SCN (10:1 by weight) at 100 A m^−^^2^ for 36 h at 298 K, using a potentiostat/galvanostat instrument (Agilent 6035A). Thermal desorption analysis was conducted to measure the hydrogen content.

The morphology and microstructure of the samples were characterized using a combination of scanning electron microscopy (SEM, JSM‐7100 F, JEOL, Japan) coupled with electron channeling contrast imaging (ECCI), energy‐dispersive spectroscopy (EDS), and transmission electron microscopy (TEM, JEOL‐2100F). TEM specimens were extracted from cross‐sections of the as‐printed and deformed gradient (CoCrMn)_100‐2x_Fe_x_Ni_x_ samples, specifically targeting the intermediate Fe_50_Ni/Co_10_Cr_10_Fe_35_Mn_10_Ni_35_/CoCrFeMnNi layer. The extracted slices were mechanically ground to a thickness of approximately 50 µm. Subsequently, 3‐mm‐diameter disks were prepared via twin‐jet electropolishing in a methanol‐based electrolyte comprising 6 vol.% perchloric acid and 34 vol.% butanol, followed by final thinning using ion‐beam milling. Electron backscatter diffraction (EBSD, Ultra 55, ZEISS) scans were performed using step sizes of 3 µm for large areas (2 mm × 1.2 mm) and 0.25 µm for small regions (< 0.5 mm × 1 mm). The samples were polished using a 0.03 lm SiO_2_ suspension. By employing AZtecCrystal 2.1 software, geometrically necessary dislocation (GND) density was deduced based on the later fine scanning step size. The grain orientation spread (GOS) map was used to characterize the microstructural conditions as recrystallized grains (GOS < 2°), substructured grains (GOS between 2° and 7°), and deformed grains (GOS > 7°). Low‐angle grain boundaries (LAGBs, 2–15°) and high‐angle grain boundaries (HAGBs, > 15°) are also indicated.

To verify the reproducibility and stability of the fabrication process, a total of 4 independent builds were fabricated under identical processing parameters. From these builds, tensile specimens were randomly selected for quasi‐static loading tests. The measured mechanical performance exhibits limited variations, confirming satisfactory inter‐batch reproducibility of the mechanical properties. In addition, three specimens from different builds were characterized by EDS and ECCI, which consistently confirmed the reproducibility of the chemical gradient, grain structure, and dislocation cell morphology across independent builds. The detailed verification results are provided in Figure S2. On the basis of this process reliability verification, the following tests and characterizations were performed on representative specimens.

### 2.3 Computational Modelling and Simulations

First‐principles calculations based on density functional theory (DFT) [26] were carried out using the exact muffin‐tin orbitals method to solve the Kohn‐Sham equations [27]. The Perdew‐Burke‐Ernzerhof generalized gradient approximation [28] was employed for the self‐consistent computation of the charge density and total energy. The intrinsic SFE was calculated, serving as relative trend indicators for the twinning effect. All calculations were performed under the assumption of a Paramagnetic (PM) or Ferromagnetic (FM) state. The PM state was described using the disordered local moment framework [29]. The coherent‐potential approximation [30] was used to model the chemical disorder. The Curie temperature (Tc) was deduced using a mean‐field approximation [31].

Molecular dynamics (MD) simulations were carried out using the LAMMPS code with periodic boundary conditions applied in all three directions. The interatomic interactions were described by the modified embedded‐atom method (MEAM) potential [32] for the Co─Cr─Fe─Mn─Ni system. This potential has been successfully employed by Seol [33] and Zhou [34] to investigate the deformation mechanism of HEA. We first constructed a nanopillar model containing 10 000 atoms (Figure S3). After obtaining a stable configuration through energy minimization, the system was equilibrated in the NPT ensemble at 300 K for 500 ps using a Nosé‐Hoover thermostat [35] and barostat [36]. To introduce chemical short‐range order, atomic models were further generated by combining Monte Carlo (MC) atom swaps with MD hybrid simulations based on the initial structures. After the MC procedure, the models were heated to 1373 K at a rate of 1 K/ps, held for 50 ps, and then cooled to the target temperatures (20, 77, 150, and 298 K) at the same rate. The nanocrystalline models of 40 Å were constructed, based on a 100‐atom unit cell generated following the same procedure as above, with 64 randomly distributed seeds selected as grain centers (Figure S4). To mitigate excessive local stresses, grain boundary atoms with nearest‐neighbor distances shorter than 1.0 Å were removed. These nanocrystalline models were then equilibrated for 600 ps in the NPT ensemble at 20 and 298 K using a Nosé‐Hoover thermostat and barostat. Uniaxial tensile loading was subsequently applied along the *z*‐axis with a strain rate of 2 × 10^8^ s^−1^ for nanopillars and 1 × 10^9^ s^−1^ for nanocrystals, using a time step of 1 fs. Owing to the limited timescales accessible to MD simulations, the applied strain rates are necessarily much higher than those used in experiments. Nevertheless, MD simulations are widely employed to investigate atomistic deformation processes and provide mechanistic insights into defect nucleation and evolution, as demonstrated in previous studies [37, 38].

Given the rapid cooling rate in the additive manufacturing process, the resultant phase constitution may differ from that predicted by equilibrium thermodynamics for the phase calculation. In this study, Scheil‐Gulliver simulations [39] were performed using Thermo‐Calc software with the TCFE2000 database and its updated version [40].

## 3 Results

### 3.1 Effect of Composition‐Induced Solidification Partition on as‐Printed Microstructures

**FIGURE 2:**
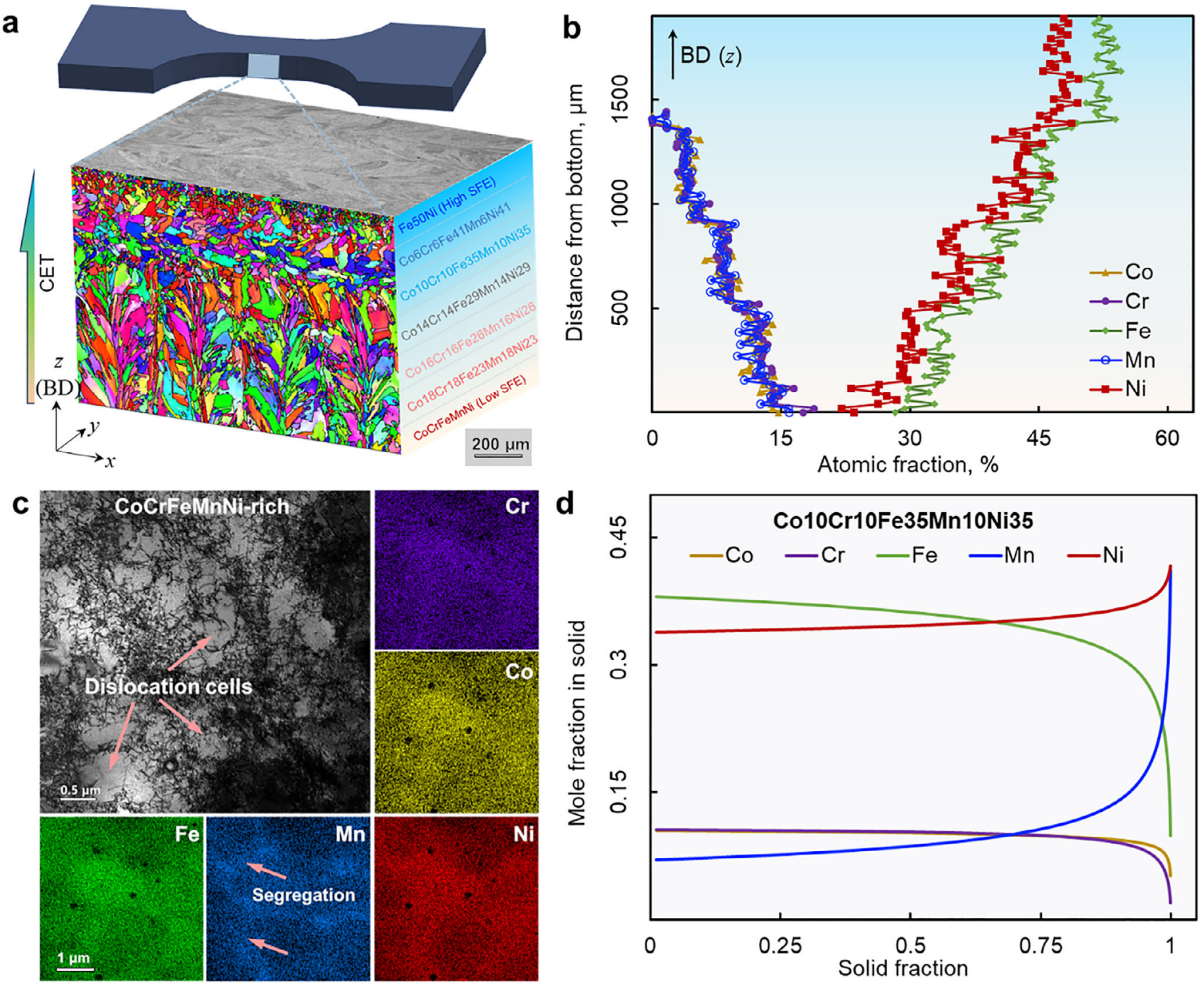
Microstructural characterizations of the in situ alloyed gradient (CoCrMn)_100‐2x_Fe_x_Ni_x_ via additive manufacturing: (a) Pseudo‐3D EBSD inverse pole figure (IPF) map of the alloy designed with schematical SFE distribution; (b) EDS map confirms the chemical gradient through the deposited layers; (c) TEM characterization coupled with EDS elemental mapping of CoCrFeMnNi‐rich region in the as‐fabricated sample; (d) Non‐equilibrium solidification phase diagram reveals the evolution of main elemental compositions in the solid phase for a typical alloy composition Co10Cr10Fe35Mn10Ni35.

Figure 2a presents a pseudo‐3D representative volume of the spatially varying microstructure in the additively manufactured gradient sample. A distinct columnar‐to‐equiaxed transition (CET) is observed from the bottom CoCrFeMnNi‐rich region to the top Fe_50_Ni‐rich region, with the transition being most pronounced within the intermediate Co_10_Cr_10_Fe_35_Mn_10_Ni_35_ layer. This CET refers to the microstructural evolution from directionally solidified columnar grains to randomly oriented equiaxed grains, a process primarily governed by the thermal gradient and constitutional undercooling ahead of the solidification front. This microstructural evolution results in a marked variation in average grain size: from 18 ± 4 µm in the Fe_50_Ni‐rich region (with a typical aspect ratio of ∼1:1.2) to 36 ± 27 µm in the CoCrFeMnNi‐rich region (typical aspect ratio ∼1:5). Moreover, the critical transition layers (Co_6_Cr_6_Fe_41_Mn_6_Ni_41_ ∼ Co_10_Cr_10_Fe_35_Mn_10_Ni_35_) consisted of ∼70% recrystallized grains, 28% substructured grains, and 2% deformed grains, as shown in Figure S5. The average geometrically necessary dislocation (GND) density was measured as 0.99 × 10^14^ m^−^^2^. Furthermore, GNDs were relatively concentrated within smaller grains, and no obvious relationship with CET was identified. The elemental mapping of Co‐Cr‐Fe‐Mn‐Ni in Figure 2b confirms the presence of a chemical gradient across the deposited layers. The Ni/Co atomic ratio increases from ∼ 1.1 near the 50 µm position to ∼ 10 near the 1200 µm position, while the Ni/Fe ratio remains relatively constant at around 0.85 throughout the gradient region. TEM characterization combined with area EDS analysis in Figure S6 reveals a dislocation cell structure with a cell size of ∼ 0.5 µm in the Fe_50_Ni‐rich region. Due to the relatively low concentrations of Cr, Co, and Mn, no significant elemental segregation is observed. In contrast, Figure 2c shows a similar dislocation cell structure on the CoCrFeMnNi‐rich region, along with noticeable segregation of Mn at the cell wall. To further quantify the local composition, EDS analyses were performed at specific layers, which are consistent with their elemental ratio in the CoCrFeMnNi‐rich region and Fe_50_Ni‐rich region, with the complete results provided in Figures S6 and S7.

For the layer‐by‐layer printed (CoCrMn)_100‐2x_Fe_x_Ni_x_ alloy, a steep cooling rate results in varying partitioning behaviors among the constituent elements [41]. To demonstrate the evolution of elements in the liquid‐to‐solid phase for a typical composition layer, the Scheil‐Gulliver model was conducted to calculate non‐equilibrium solidification. As shown in Figure 2d of the selected transition layer Co_10_Cr_10_Fe_35_Mn_10_Ni_35_, prior to final solidification, the concentrations of Co, Fe, and Cr decrease in the liquid phase, while Mn exhibits a pronounced tendency for segregation. This corresponds well with EDS analysis in Figure 2c. Moreover, Fe and Ni display opposing partitioning trends within the solid phase. Additional phase diagram calculations for intermediate compositional layers are provided in Figure S7. As the composition shifts from the equiatomic CoCrFeMnNi toward the Fe_50_Ni layer, the effect on Mn segregation diminishes. Concurrently, the distribution of Fe and Ni in the solid phase begins to partition together. The CET shown in Figure 2a is attributed to this element partitioning behavior [42, 43].

### 3.2 Quasi‐static Tensile Response at Cryogenic to Ambient Temperatures

**FIGURE 3:**
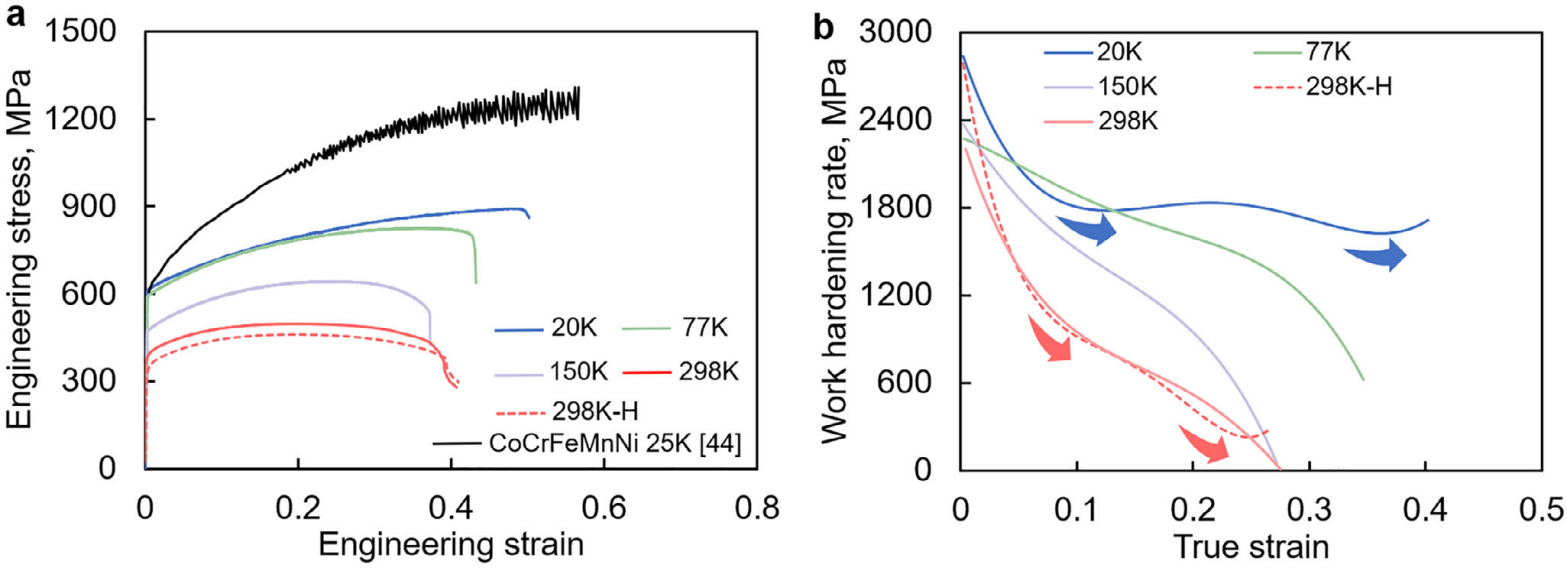
(a) Engineering stress‐strain curves of gradient samples and reference CoCrFeMnNi with DPF under 25K [44] under various temperatures with quasi‐static tensile deformation; (b) Work hardening rate of corresponding samples. The blue and red arrows point to the upturns of the hardening rate.

Figure 3a illustrates the tensile mechanical properties of the gradient (CoCrMn)_100‐2x_Fe_x_Ni_x_ samples at a slow strain rate under various temperatures. As the temperature decreased from 298 to 20 K, both the yield strength (YS) and ultimate tensile strength (UTS) increased significantly. The YS raises from 356 ± 4 MPa to 614 ± 9 MPa, while the UTS increased from 498 ± 8 MPa to 891 ± 12 MPa. Total elongation (TE) remained consistently at a high level, ranging between 37.2% and 43.2%, with no clear monotonic trend. The mechanical properties under different temperatures are summarized in Table S1.

In contrast to the pronounced stress drop ∼ 40 MPa reported for homogeneous CoCrFeMnNi at 35–45% cryogenic tensile strain [44], the gradient (CoCrMn)_100‐2x_Fe_x_Ni_x_ demonstrates negligible stress drop during deformation at 20 K, as shown in Figure 3a. Magnified views of the tensile stress‐strain curves for specimens tested at 77, 150, 298 K, and hydrogen‐charged at 298 K are provided in Figure S8, highlighting that the serrated stress with DPF persists at cryogenic temperatures.

The strain hardening rates of the different samples are presented in Figure 3b. Under slow strain‐rate tensile conditions from 298 K down to 77 K, the hardening rate in all cases starts at approximately 2200 MPa, and then declines rapidly initially, followed by a more gradual reduction, and finally a rapid drop. In contrast, both the hydrogen‐charged specimen and the specimen tested at 20 K exhibit initial strain hardening rates of around 2800 MPa, which decrease with increasing true strain. Notably, at 20 K, the hardening rate remains above 1800 MPa throughout the deformation process, indicating sustained dislocation‐mediated hardening capability.

In the hydrogen‐charged condition tested at 298 K, the YS was 323.1 MPa, and the UTS was 460.3 MPa, with a TE of 39.4%. Notably, the hydrogen‐charged specimen exhibited virtually no loss in ductility compared to the uncharged sample, despite a reduction in strength of approximately 32.6 MPa in YS. The retention of ductility is critical for structural materials to resist HE, as it prevents the rapid propagation of hydrogen‐induced cracks, a concern to which additively manufactured materials are particularly susceptible due to the presence of microcracks or pores. Furthermore, analogous to the work hardening rate curve observed at 20 K, the hydrogen‐charged specimens also exhibited two distinct upturns during deformation, as indicated by the arrows, suggesting a transition in deformation mechanisms.

Therefore, a better balance between cryogenic plastic stability and hydrogen tolerance of the gradient (CoCrMn)_100‐2x_Fe_x_Ni_x_ is achieved.

## 4 Discussion

### 4.1 Microstructure‐Mediated Basic Deformation Response at Ambient Temperature

The processing‐induced solidification conditions and compositional undercooling vary across different chemical zones in the deposition section, as revealed by the thermodynamic simulations in Figure S9. A gradient microstructure in the as‐fabricated (CoCrMn)_100‐2x_Fe_x_Ni_x_ sample in Figure 1 confirms the CET feature. This meso‐structure, in turn, dictates the deformation mechanics. The compositional gradient ensures interfacial compatibility and suppresses strain localization through activating dislocation slip and twinning [23, 45], thereby reducing deformation concentration, as also validated by post‐deformation analysis in Figure S10.

**FIGURE 4:**
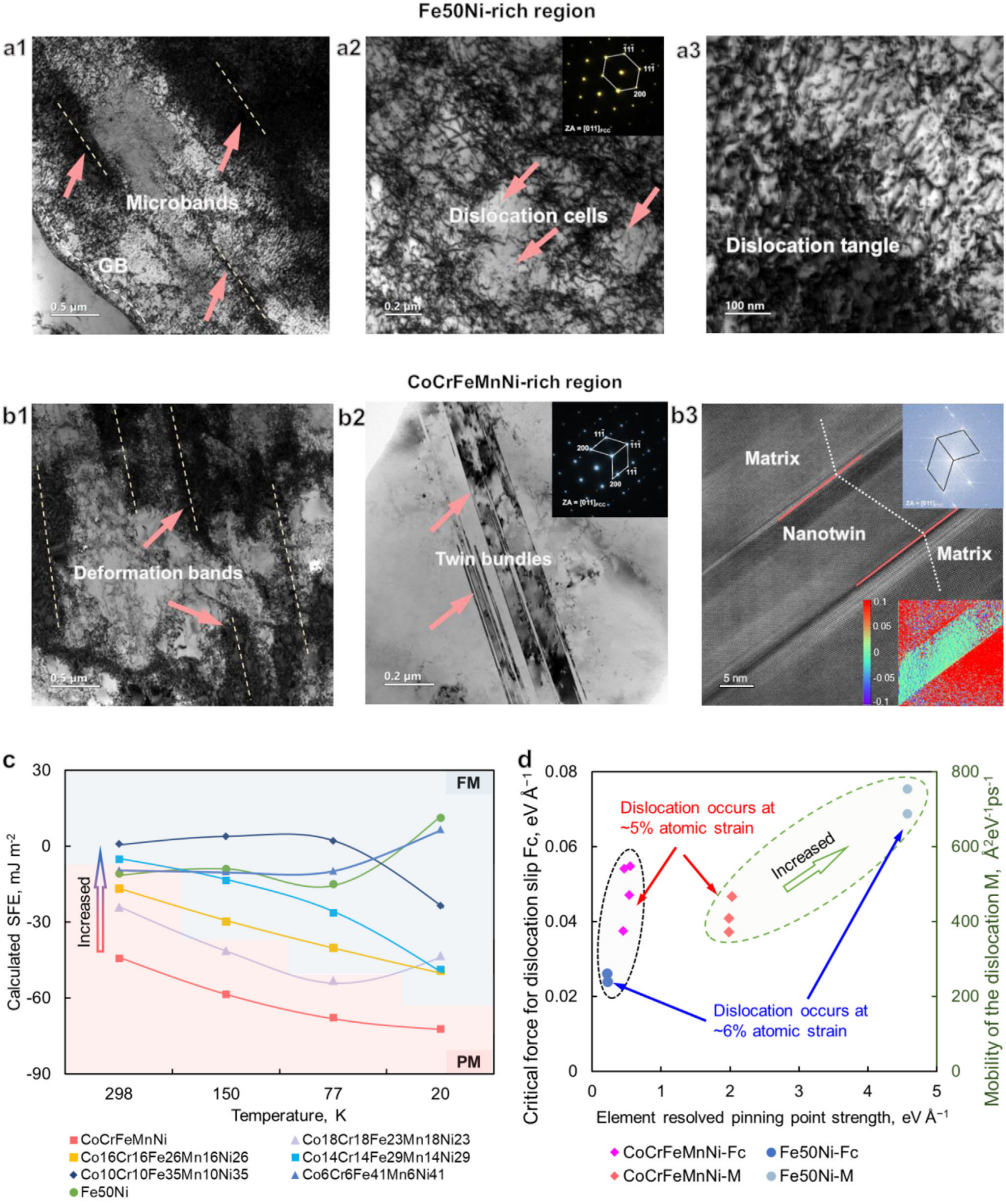
Comparative TEM analysis at different positions of a deformed sample at 298 K: (a) Fe_50_Ni‐rich region and (b) CoCrFeMnNi‐rich region. In the Fe_50_Ni‐rich region with high SFE, dislocations primarily undergo cross‐slip within grains, preserving a cellular substructure and distinct dislocation lines. In contrast, planar slip dominates in the low‐SFE CoCrFeMnNi‐rich region, leading to the formation of (b1) dislocation walls and (b2) Deformation twin bundles. (b3) Incipient twin lamellae with a width of ∼ 20 nm with GPA analysis. (c) SFEs of representative compositions estimated by MD simulations with corresponding FM/PM states derived from DFT calculations (Table S2). Note that the negative SFE values obtained from DFT calculations do not physically imply that the material will spontaneously generate stacking faults; rather, they serve only as relative trend indicators. (d) Atomic tensile strain corresponding to dislocation nucleation in model alloys Fe_50_Ni and CoCrFeMnNi, analyzed using slip resistance metrics as critical force for dislocation slip Fc and mobility of dislocation M adopted in Ref. [53] by MD simulations.

A comparative TEM analysis is conducted on the deformed microstructures near the Fe_50_Ni‐rich and CoCrFeMnNi‐rich regions, as shown in Figure 4. Owing to its higher SFE, dislocation cross‐slip and strong entanglement are prevalent in the Fe_50_Ni‐rich region and preserve cellular substructure with distinct dislocation lines, as shown in Figure 4a. In contrast, planar slip dominates in the CoCrFeMnNi‐rich region due to its lower SFE, resulting in the formation of distinct slip bands [46, 47]. Additionally, nanoscale twin bundles are observed in Figure 4b2, accompanied by the corresponding selected‐area electron diffraction (SAED) pattern. Figure S11 further reveals the appearance of stacking faults (SFs) in the CoCrFeMnNi‐rich region. High‐resolution TEM (HR‐TEM) image of Figure 4b3 reveals nanoscale twins with a width of approximately 20 nm. Geometric phase analysis (GPA) indicates that localized strain is predominantly concentrated at the interfaces between the matrix and twin boundaries.

To understand inherent differences in SFE across compositional layers, Table S2 presents the intrinsic SFEs predicted by DFT calculations for seven typical atomic ratio combinations under both ferromagnetic (FM, i.e., a magnetically ordered state with spontaneous alignment of magnetic moments) and paramagnetic (PM, i.e., a magnetically disordered state where thermal fluctuations destroy long‐range order) conditions, as well as the deduced Curie temperatures (Tc, the critical temperature at which the FM‐PM phase transition occurs). At a temperature range of 20–150 K, layers with higher Co, Cr, and Mn content exhibit relatively lower SFEs, as shown in Figure 4c. These layers are more prone to twin formation at cryogenic temperatures [48, 49]. The Co_10_Cr_10_Fe_35_Mn_10_Ni_35_ and Fe_50_Ni possess a medium‐to‐high intrinsic SFE.

Variations in chemical composition alter interatomic bonding forces and atomic arrangements, thereby influencing crystal lattice stability [50, 51, 52]. For comparison of chemical element‐mediated and temperature‐dependent deformation behavior of the gradient alloy, MD simulations for uniaxial tensile on typical single‐layered Fe_50_Ni, Co_10_Cr_10_Fe_35_Mn_10_Ni_35_, and CoCrFeMnNi are conducted at 20 and 298 K. As seen in Figure S3, the YS increases with decreasing temperature, while progressively decreasing among the three compositions, in accordance with their respective SFE sequence. For specific compositions such as Co_18_Cr_18_Fe_23_Mn_18_Ni_23_, Co_6_Cr_6_Fe_41_Mn_6_Ni_41_, and Fe_50_Ni, the SFE exhibits an anomalous increase when the temperature is lowered from 77 to 20 K.

As summarized in Figure 4d, the critical force for dislocation slip of CoCrFeMnNi HEA is higher than Fe_50_Ni and their mobility of dislocation are opposite [53]. The CoCrFeMnNi and Fe_50_Ni alloy systems exhibit a marked sensitivity to the Peierls barrier height [53, 54]. The local distribution of Peierls stress and its effect on dislocation pinning forces enhance the material's resistance to the initiation of dislocation slip and reduce the overall dislocation mobility.

### 4.2 Suppressed Discontinuous Plastic Flow at Cryogenic Temperatures

**FIGURE 5:**
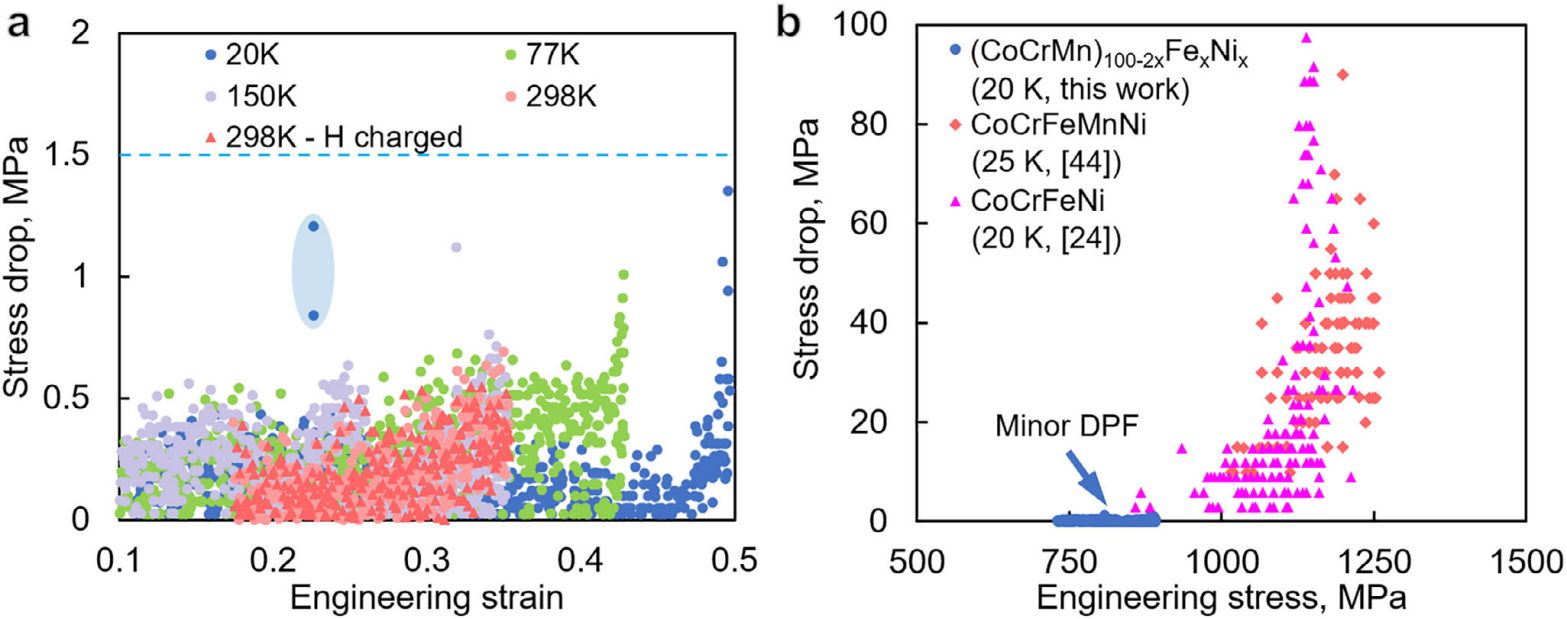
(a) The temperature‐variant stress drop is plotted as a function of engineering strain. Suppressed DPF with minor stress drops below 1.5 MPa for (CoCrMn)_100‐2x_Fe_x_Ni_x_; (b) Comparison on stress drops of CoCrFeMnNi [44], CoCrFeNi [24], and (CoCrMn)_100‐2x_Fe_x_Ni_x_ at ∼ 20 K.

Figure 5 plots the temperature‐variant stress drop against engineering strain for the (CoCrMn)_100‐2x_Fe_x_Ni_x_ alloys, which exhibit suppressed DPF with amplitudes below 1.5 MPa, and compares these values with CoCrFeMnNi [44] and CoCrFeNi [24] at ∼20 K. At cryogenic temperature range of 20–77 K, effectively suppressed DPF is associated with minor stress drops of 0.1–1.5 MPa, far less than 0.1% of the YS at 298K (∼335 MPa), as demonstrated in Figure 5a. With increasing plastic deformation and decreasing temperature, the amplitude of the stress fluctuations generally diminishes. Figure S8 compares the DPF behavior at ∼ 22% tensile strain, further revealing that the negligible stress fluctuations in the later stages of deformation via variant conditions. Such suppressed DPF contributes to enhanced service reliability.

Compared to homogeneous CoCrFeMnNi [44] and CoCrFeNi [24], the stress drops in the current gradient sample are relatively tiny (Figure 5b). The average grain size in the printed gradient alloy ranges from 18 ± 4 µm in the Fe_50_Ni‐rich region to 36 ± 27 µm in the CoCrFeMnNi‐rich region, which is comparable to or slightly finer than the grain sizes reported in the reference studies (typically 20 – 50 µm for the homogeneous HEAs).

In classical DPF theory, lower strain rates promote more complete pinning–depinning cycles of dislocations, thereby intensifying serrated flow [5, 6]. Here, a strain rate of 1 × 10^−4^ s^−1^ is employed, which falls within the typical DPF regime of 5 × 10^−5^ s^−1^ ∼ 8.5 × 10^−3^ s^−1^ [5, 6, 24, 44] and is lower than the rates applied to homogeneous CoCrFeMnNi [44] and CoCrFeNi [24], enhancing the reliability of the gradient (CoCrMn)_100‐2x_Fe_x_Ni_x_ alloy under cryogenic quasi‐static loading. This intermediate rate was deliberately selected to also meet the slow tensile testing requirements for hydrogen‐charged specimens (Section 4.4), facilitating a unified evaluation of cryogenic plastic flow and ambient‐temperature hydrogen‐assisted deformation behavior. Notably, the present gradient alloy exhibits stress‐drop amplitudes an order of magnitude smaller than those reported for those reported at higher strain rates (1 × 10^−3^ s^−1^) [24, 44].

While variations in specimen geometry and system compliance may also contribute quantitatively to stress‐fluctuation characteristics, the observed reduction far exceeds reasonable systematic errors tested at ambient temperature (Figure S8), confirming that the gradient design fundamentally suppresses DPF.

The pinning agents here are intrinsic crystal defects rather than solute atoms, as entirely determined by dislocation density that directly triggers serrated flow [55, 56, 57]. This cryogenic DPF is attributed to the pinning of mobile dislocations by immovable dislocation locking substructures, SFs, and cross twins at cryogenic temperatures [6, 44, 58]. Therefore, increasing the SFE fundamentally suppresses unconventional deformation mechanisms, reducing effective pinning points for dislocations and consequently weakening or even eliminating serrated flow. In addition, the additive manufacturing process introduces a high density of uniformly distributed and entangled dislocations within the material, forming stable dislocation cellular structures (Figure 3). This pre‐existing dislocation network can “disrupt” the local pinning processes, dominated by twins and SFs, that serrated flow relies on during subsequent cryogenic deformation. As a result, deformation is channeled through the pre‐existing network, which may inhibit the initiation of serrations. Concurrently, the cellular dislocation substructures and substructured grains induced by the DED process serve as pre‐existing pinning sites that compete with deformation‐induced twins and SFs, thereby disrupting the avalanche‐like pinning‐depinning cycles characteristic of classical DPF. Furthermore, the deliberate design of SFE modulates the dislocation slip mode and associated substructures, thereby influencing this mechanism, further corroborated by MD simulations in the next Section.

### 4.3 Mechanically Activated Gradient Twinning at Wide‐range Temperatures

**FIGURE 6:**
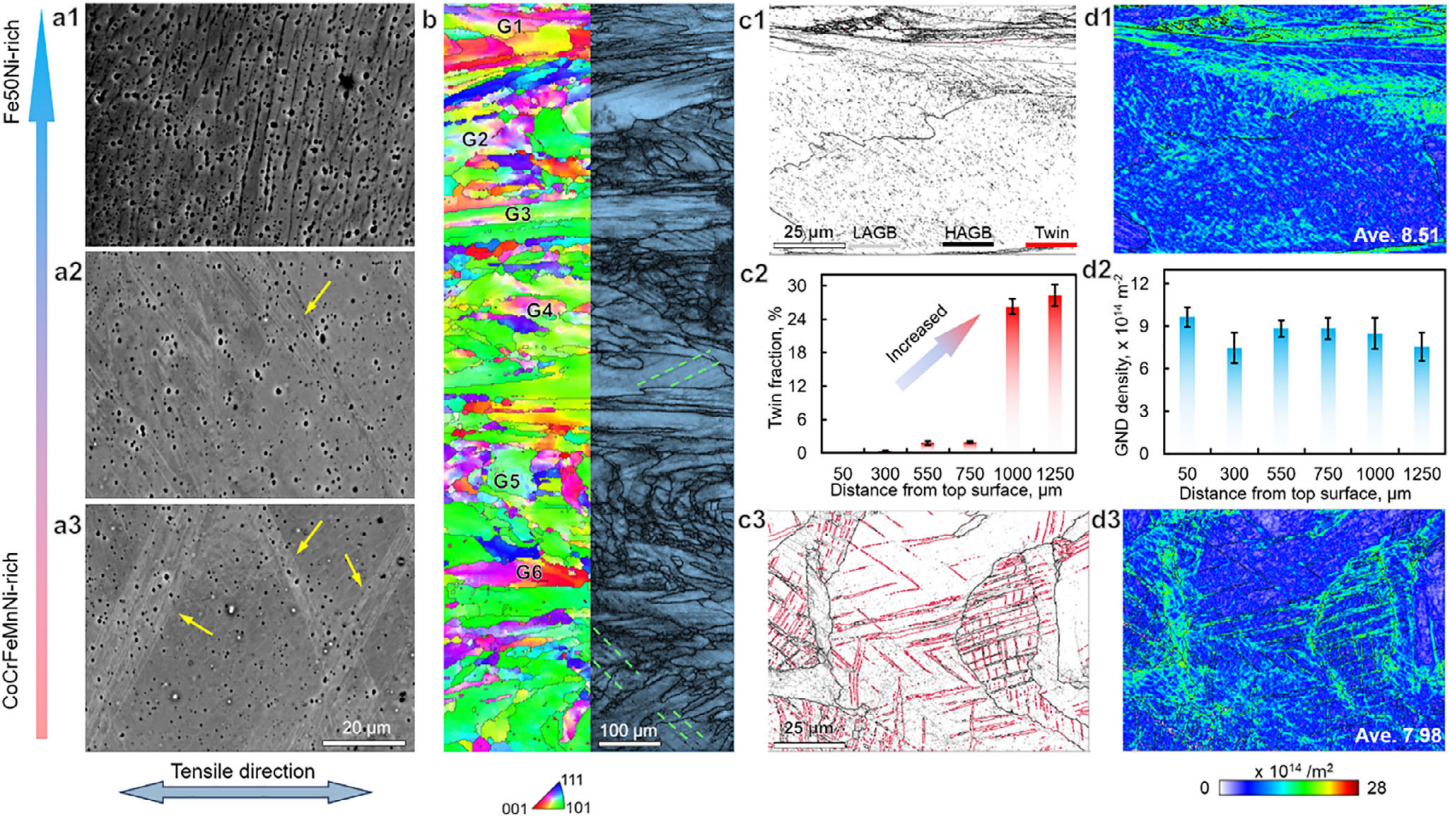
Deformation microstructure and gradient twin distribution of the gradient (CoCrMn)_100‐2x_Fe_x_Ni_x_ sample tensile‐tested at 20 K. (a) ECCI images revealing distinct dislocation slip and nano‐twinning modes in three regions: (a1) High‐SFE Fe_50_Ni‐rich region, (a2) Intermediate composition transition zone, and (a3) Low‐SFE CoCrFeMnNi‐rich region. (b) EBSD IPF map showing grains elongated along the tensile axis. The dashed lines in the right‐part band contrast map denote the micro‐deformation bands. Local deformation analysis: (c‐d) Fe_50_Ni‐rich region with (c1) Band contrast and (d1) GND distribution map (average density: 8.51 × 10^14^ m^−2^) illustrating cross‐grain slip traces; CoCrFeMnNi‐rich region with (c3) Band contrast and (d3) GND map (average: 7.98 × 10^14^ m^−2^) showing intra‐granular slips and nanotwins. Statistical profiles: (c2) Twin boundary fraction and (d2) Average GND density, both plotted against distance from the Fe_50_Ni‐rich region surface, quantifying the gradient twin structure and strain hardening behavior.

Figure 6 presents the deformed microstructure of the (CoCrMn)_100‐2x_Fe_x_Ni_x_ sample at 20 K. ECCI taken from near the fracture surface (Figure 6a) reveals elongated cellular structures in the high‐SFE Fe_50_Ni‐rich region (Figure 6a1), while distinct slip band markings are observed at the intermediate location (Figure 6a2). In the low‐SFE CoCrFeMnNi‐rich region, multiple intersecting slip bands are evident (Figure 6a3). IPF map in Figure 6b covering the entire graded cross‐section further confirms the presence of elongated deformed grain structures. Deviated orientations are detected within grains despite of SFE levels (e.g., G1–G6), indicative of substantial lattice distortion by local dislocation rearrangement [59, 60]. The grain boundaries are highlighted in Figure 6c1,c2, revealing a gradient twin boundary trend from the high‐SFE Fe_50_Ni‐rich side to the low‐SFE CoCrFeMnNi‐rich region. As statistically summarized in Figure 6c3, the twin boundary fraction increases significantly with increasing distance from the surface high‐SFE Fe_50_Ni‐rich region.

Figure 6d1,d2 display the gradient in GND density. Interestingly, the average GND density does not show correlation with the SFE gradient, as quantified in Figure 6d3. This can be attributed to high‐angle grain boundaries and twin boundaries that act as effective barriers to GND accumulation [61, 62, 63]. In the present design, these features facilitate improved dynamic strain compatibility between layers of different SFEs through the gradient composition tailoring from CoCrFeMnNi‐rich to Fe_50_Ni‐rich regions.

**FIGURE 7:**
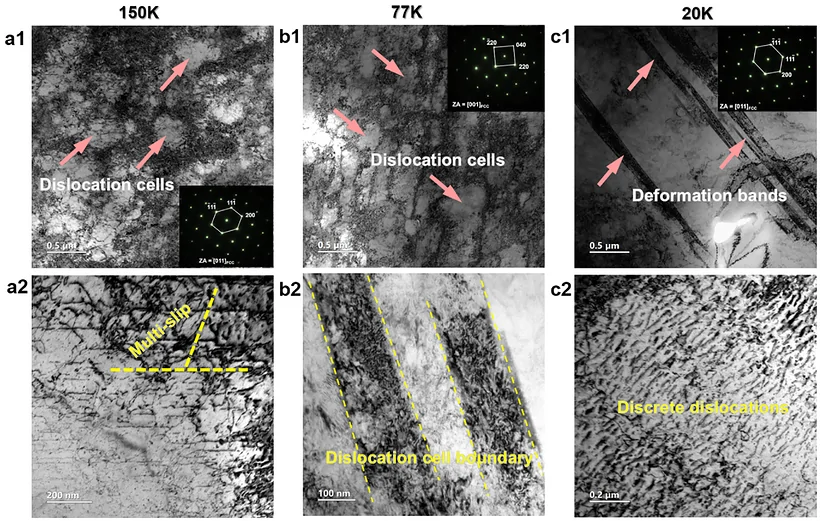
Enhanced dislocation planarity and absence of twinning in the Fe_50_Ni‐rich region during cryogenic deformation. Selected‐zone TEM images show microstructures at 20, 77, and 150 K at different magnifications to reveal the typical substructures: (a1) Dislocation cells, (b1) Sharpening dislocation cells, (c1) Deformation bands. Inserted SAED patterns verify no twin or phase transformation. (a2) Long dislocation slip lines, (b2) Cell wall detail, (c2) Discrete short dislocation slip. Scale bar differentiation between rows highlights the dislocation features under identical temperature conditions.

In contrast, the Fe_50_Ni‐rich region does not exhibit twinning, which can be attributed to relatively high SFE across the temperature range [64]. The SFE of Fe_50_Ni also decreases with decreasing temperature. During plastic deformation, the dominant dislocation mechanism transitions from a mixed mode of cross‐slip and planar slip, manifested as the wavy slip shown in Figure 7a2, toward pronounced planar slip (Figure 7a1–b1). SAED patterns confirm the absence of both twinning and martensitic transformation even at cryogenic temperatures. Concurrently, well‐defined dislocation cell walls change into deformation bands, as observed in Figure 7b1–c1. As planar slip accumulates and temperature decreases, dislocation features such as Taylor lattices and microbands are activated, contributing to an enhanced work‐hardening capacity [65, 66].

**FIGURE 8:**
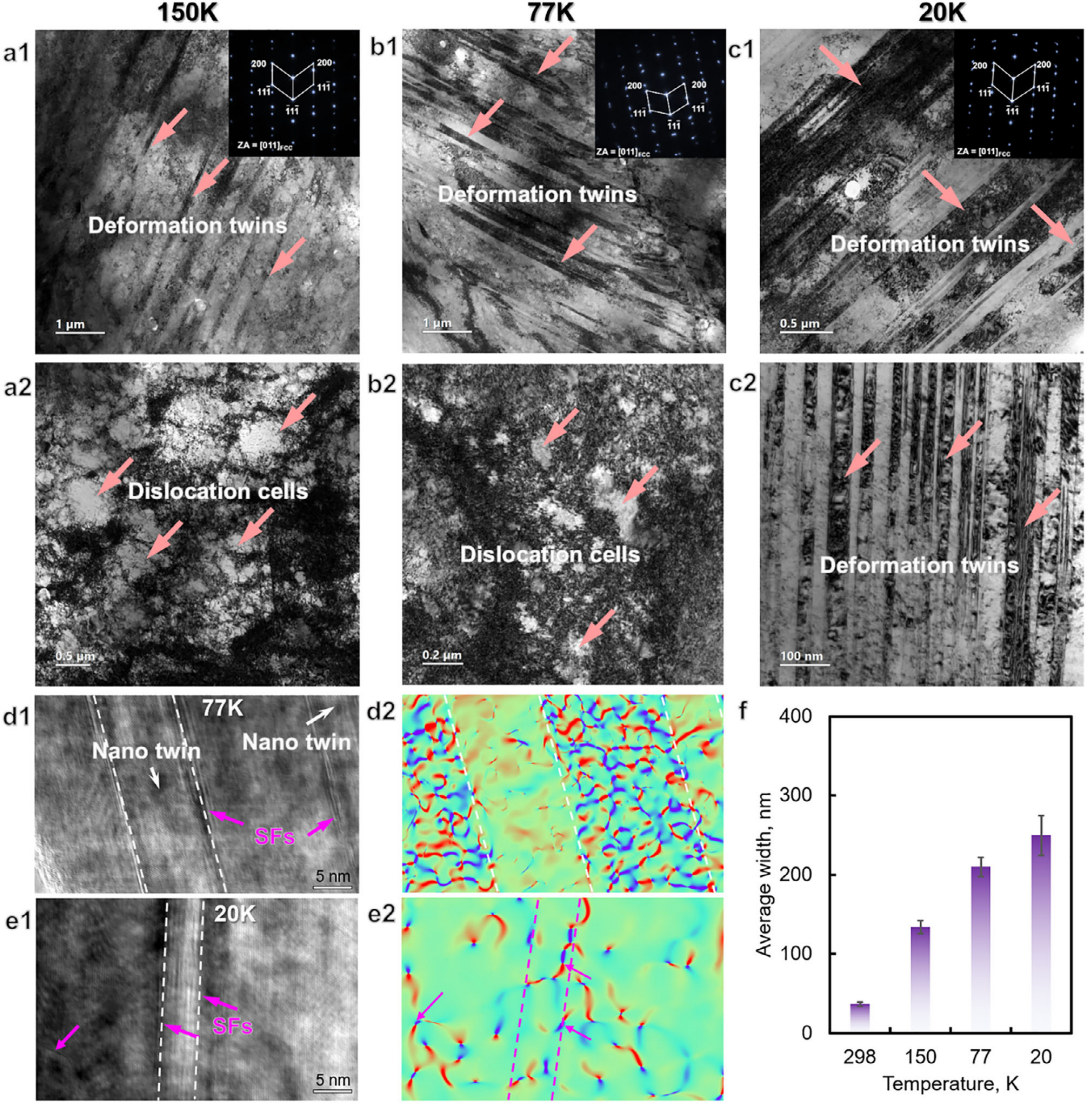
Deformation microstructures in the CoCrFeMnNi‐rich region at cryogenic temperatures (20, 77, and 150 K). SAED patterns confirm the absence of martensitic transformation and the presence of deformation twins with high dislocation density within nano‐twins. (a)‐(c) Bright‐field images at 150, 77, and 20 K, respectively. HR‐TEM images on deformed samples with GPA analysis on lattice strain at (d) 77 K and (e) 20 K. (f) Statistical analysis of average twin width across the cryogenic‐to‐ambient temperature range.

Moreover, in the CoCrFeMnNi‐rich region, the SFE exhibits a marked decrease with declining temperature [67], leading to a promotion for twinning during plastic deformation. At 150 K, nanotwins and dislocation cell structures are evident, as observed in Figure 8a1,a2. With further temperature reduction to 77 and 20 K, both the density and width of the twins increase significantly, as shown in Figure 8b1,c2,c3. In low‐SFE materials, the extensive spreading of dislocations restricts their movement to narrow slip bands [68], promoting strong dislocation interactions (e.g., entanglement and reactions) and leading to strain localization, which fundamentally accounts for their enhanced work hardening rate. At lower temperatures, dislocation cross‐slip are suppressed. Consequently, dislocations accumulate, resulting in a notable increase in dislocation density (Figure 8a2 vs Figure 8b2).

HR‐TEM images in Figure 8d1,e1 further present the nanotwin and SFs at 77 and 20K. The misorientation between the twins and the matrix, combined with stress concentration caused by dislocation pile‐ups, places the twin boundary under a locally high‐stress state, as proved in the GPA image in Figure 8d2. This high stress provides a driving force for activation of dislocation sources within the twins, thereby efficiently generating a high density of dislocations near the twins. SFs also cause local lattice strain mismatch according to the GPA analysis of Figure 8e2. The variation in average nanotwin width across 20 to 298 K is summarized in Figure 8f. At ambient temperature, the local stress rarely reaches the critical twinning stress (∼720 MPa) [69]. Consequently, only a limited number of ultrafine nanotwins (average thickness: 37 ± 2.8 nm) are observed in the CoCrFeMnNi‐rich region. In contrast, at 77 and 20 K, twinning becomes prevalent, with average twin thicknesses of 204 ± 12 nm and 250 ± 25 nm, respectively. This trend results from the strong suppression of dislocation cross‐slip, causing dislocations to accumulate on slip planes and generate significant local stress concentrations. Consequently, this high local stress provides a potent driving force for twin thickening. It facilitates the nucleation and multiplication of partial dislocations at the twin boundaries, a process that mediates the lateral growth of the twins and increases their average width.

**FIGURE 9:**
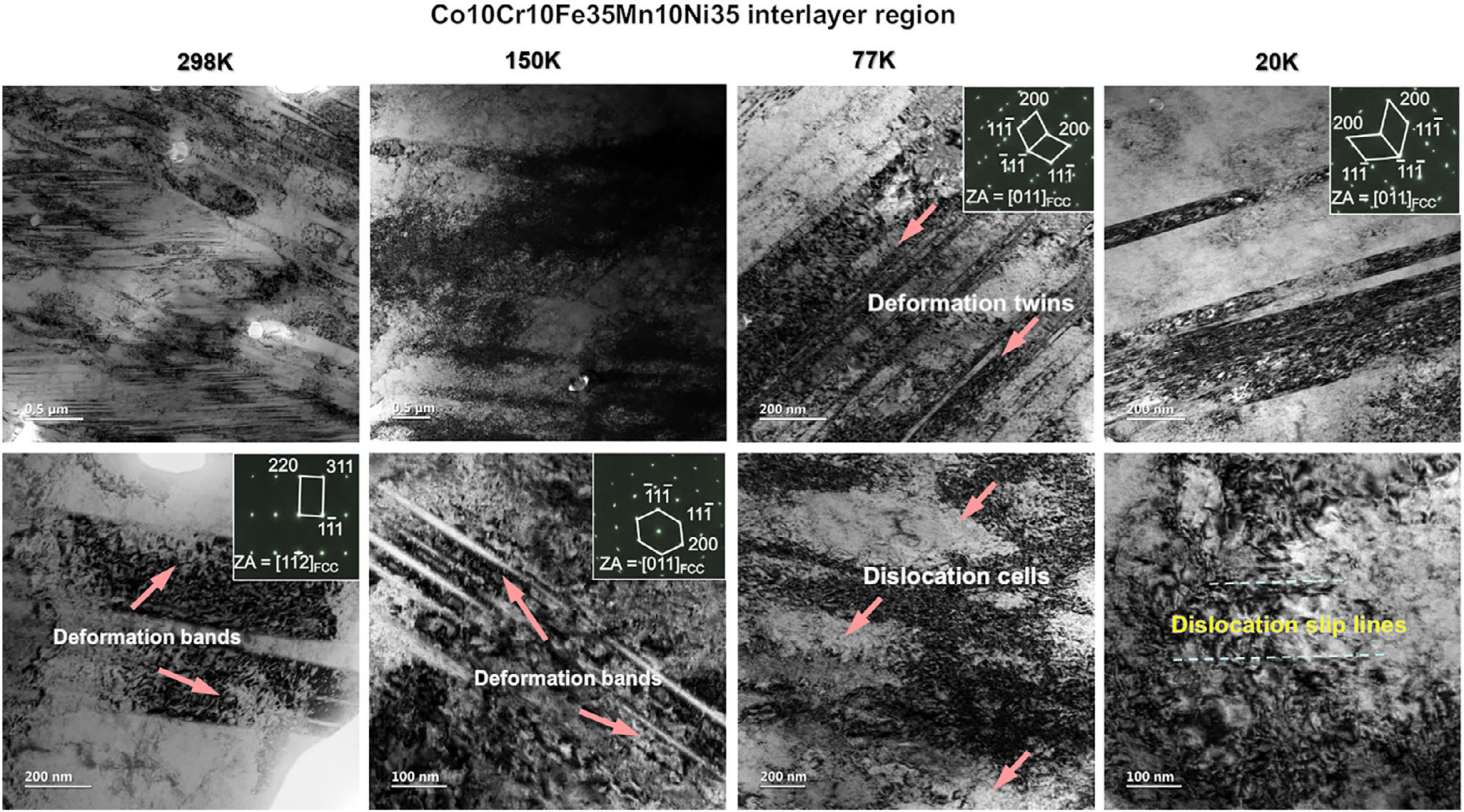
TEM characterizations on the nominal Co_10_Cr_10_Fe_35_Mn_10_Ni_35_ interlayer region after tensile straining at 298, 150, 77, and 20 K. At cryogenic temperatures, the deformed samples exhibit limited twin formation with SAED patterns.

Moreover, as evidenced by Figure 9 and Figure S12, the intermediate Co_10_Cr_10_Fe_35_Mn_10_Ni_35_ layer exhibited limited twinning activity upon tensile straining at 77 K and even at 20 K. Its deformation characteristics demonstrate a mixed mode that bridges the Fe_50_Ni‐rich region (Figure 7c) and the CoCrFeMnNi‐rich region (Figure 8c). The cryogenic evolution of deformation twinning correlates well with the temperature dependence of the intrinsic SFE for this intermediate layer, as determined by DFT calculations in Figure 4c. From a compositional design perspective, the intermixing of CoCrFeMnNi and Fe_50_Ni in equal proportions facilitates a smooth gradient transition in both SFE and chemical composition. With decreasing deformation temperature, the dislocation slip modes progressively transition from cross‐slip to planar slip within the respective constituent phases, accompanied by thickening of the twins. Consequently, this gradient configuration enhances deformation delocalization [70, 71], thereby significantly improving the cryogenic deformation compatibility of the alloy.

The gradient alloy with a broad distribution of SFE leads to localized peaks of frictional strengthening within the energy landscape. Particularly at cryogenic temperatures, partial dislocations must overcome these local energy barriers during slip [72]. Furthermore, the interaction between Co and Cr leads to the densest distribution of potent dislocation pinning sites in the CoCrFeMnNi‐rich region [53, 54]. This mechanism is responsible for the DPF effect observed in the CoCrFeMnNi HEA at low temperatures [57].

**FIGURE 10:**
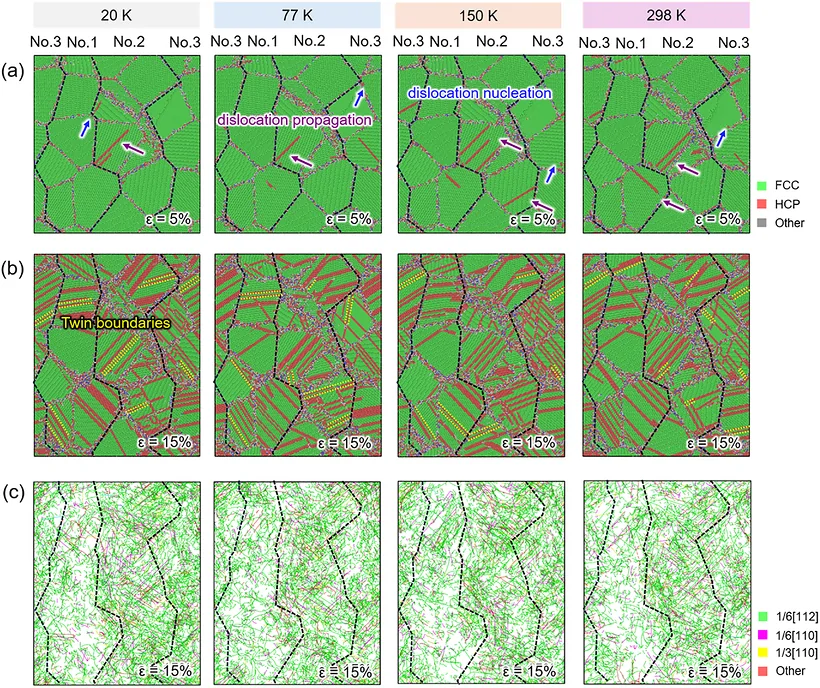
(a, b) Representative atomic configurations of the nanolaminate model deformed to tensile strains of (a) 5% and (b) 15% at 20, 77, 150, and 298 K. Atoms are colored according to their local structural environments: FCC (green), HCP (red), and other structures (gray). (c) Dislocation configurations extracted at a tensile strain of 15%.

Moreover, Figure 10 and Figure S4 show that temperature has a relatively minor influence on dislocation density in nano‐laminated Fe_50_Ni (No. 1) / Co_10_Cr_10_Fe_35_Mn_10_Ni_35_ (No. 2) / CoCrFeMnNi (No. 3) of fixed composition, particularly in the high‐SFE Fe_50_Ni layer. In contrast, compositional effects are more pronounced: layers with medium‐to‐low SFE that are enriched in Co, Cr, and Mn display a rapid increase in dislocation density with strain, reaching higher values at 20 K than at 298 K. Since SFs and twins within grains are formed through dislocation‐mediated processes, a higher dislocation density consequently promotes their formation.

Figure 10a,b present representative microstructures of the nanolaminate model at tensile strains of 5% and 15% at different temperatures. At a strain of 5%, dislocation nucleation and glide are already evident; as the strain increases to 15%, extensive dislocation glide and accumulation occur within the constituent layers, accompanied by the formation of pronounced SF‐ and twin‐related structures. The red atoms primarily represent local HCP environments generated by the glide of Shockley partial dislocations and therefore indicate planar defects such as SFs and twin boundaries within the FCC matrix. Combined with the evolution of dislocation density shown in Figure 10c, these results might help explain the pronounced differences in dislocation activity among the constituent layers: the Co‐, Cr‐, and Mn‐rich layers with medium‐to‐low SFEs exhibit higher dislocation densities and more active dislocation evolution, whereas dislocation accumulation is comparatively limited in the high‐SFE Fe_50_Ni layer. In FCC alloys, the glide of Shockley partial dislocations generates SFs, whereas the coordinated glide of twinning partials on successive {111} planes facilitates the nucleation and growth of deformation twins [73, 74]. Consequently, the enhanced partial‐dislocation activity in the medium‐ and low‐SFE layers promotes SF accumulation and the successive passage of partial dislocations on adjacent crystallographic planes, thereby increasing the propensity for nanotwin formation. This composition‐dependent trend in defect evolution simulated by MD is generally consistent with the experimental observation that nanotwins preferentially form in the low‐SFE regions enriched in Co, Cr, and Mn.

It should be emphasized that the atomistic simulations and experiments operate at substantially different strain rates and length scales. The high MD strain rate can shift defect‐nucleation stresses and strains, restrict thermally activated relaxation, and alter the competition among dislocation glide, cross‐slip, and twinning. Therefore, the MD configurations are interpreted only in terms of relative trends among the compositional layers, rather than as a direct reproduction of the experimental deformation sequence. The experimentally observed dislocation and twin structures provide the primary evidence for the proposed deformation mechanisms, whereas the MD results offer atomistic‐level support for how the composition‐ and SFE‐dependent differences may develop.

The deformation response of the graded alloy is strongly temperature‐dependent: at ambient temperature, wavy slip prevails, whereas under cryogenic conditions, planar slip dominates, accompanied by possible deformation twinning in the CoCrFeMnNi‐rich regions, while the Fe_50_Ni‐rich layers sustain multi‐system dislocation slip across the entire temperature range. As summarized in Table 1, this behavior originates from the temperature‐adaptive SFE gradient (Figure 4c), which leads to a graded activation of slip systems. Specifically, the high‐SFE Fe_50_Ni‐rich regions promote cross‐slip and enable dislocation motion on multiple planes at cryogenic temperatures (Figure 7), effectively redistributing strain and preventing local stress concentrations. In contrast, the low‐SFE CoCrFeMnNi‐rich regions favor planar slip; however, the adjacent high‐SFE layers partially accommodate the plastic strain, thereby suppressing uncontrolled planar slip that would otherwise cause avalanche‐like dislocation activity. Additionally, geometrically necessary boundaries and twin boundaries in the low‐SFE zones serve as dislocation sinks that relieve local stress accumulation, as corroborated by MD simulations (Figure 10 and Figure S4). The cooperative interplay between the high‐ and low‐SFE regions effectively balances slip planarity and multiplicity across the graded architecture, enhancing cryogenic deformation compatibility and successfully suppressing DPF.

**TABLE 1:** Summary of local composition, DFT‐predicted SFE, and corresponding deformation mechanisms for selected gradient layers along with temperature decrease.

Layer designation	DFT‐predicted SFE (mJ/m^2^)	Dominant deformation mechanism	Microstructural features at decreasing temperatures
Fe_50_Ni rich	∼ 100 (FM) / ∼ 72 (PM)	Wavy slip / cross‐slip	Dislocation cells, sharpened cell walls, deformation bands
Co_10_Cr_10_Fe_35_Mn_10_Ni_35_ (Interlayer)	∼ 36 (FM) / ∼ 36 (PM)	Transitional slip	Mixed dislocation cells and limited twin formation
CoCrFeMnNi‐rich	∼ ‐56 (FM) / ∼ ‐72 (PM)	Planar slip + nanotwinning	Planar slip bands, SFs, deformation twin bundles

*Note*: DFT calculations are utilized to interpret the trends associated with compositional variations (Table S2). In this regard, the negative SFE values derived from DFT serve only as qualitative indicators of relative ordering and should not be regarded as absolute physical values.

### 4.4 Origin of Hydrogen Embrittlement Resistance

**FIGURE 11:**
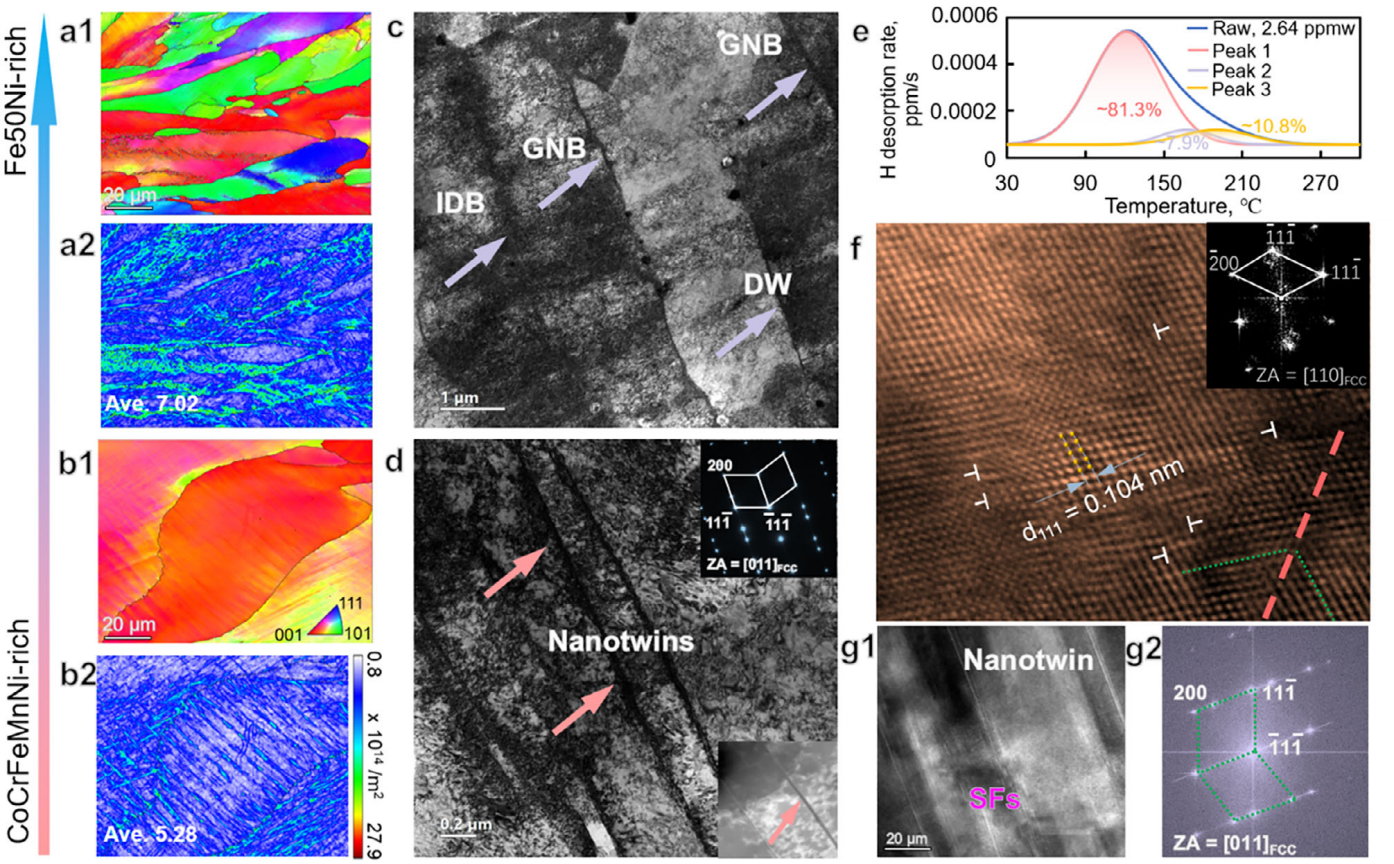
Characterizations of deformed H‐charged gradient (CoCrMn)_100‐2x_Fe_x_Ni_x_ sample. (a) IPF and GND of Fe_50_Ni‐rich region; (b) IPF and GND of CoCrFeMnNi‐rich region; (c) TEM observation of Fe_50_Ni‐rich side; (d) TEM observation of CoCrFeMnNi‐rich region; (e) The TDA plot of the dog‐bone sample, showing the raw data curve and three deconvoluted peaks; (f) Atomic phase observation of CoCrFeMnNi‐rich region showing lattice distortion with nanotwin boundary; (g) HR‐TEM observation of CoCrFeMnNi‐rich region with SAED pattern, revealing the nanotwin and SFs.

In the graded (CoCrMn)_100‐2x_Fe_x_Ni_x_ alloy, resistance to HE is achieved (Figure 3a) through a dual‐protection mechanism consisting of a graded Mn distribution and deformation‐induced nanotwins (Figure 6c2). This mechanism is preferentially activated in the CoCrFeMnNi‐rich region, as confirmed by the gradient nanotwin structure observed in Figure 11a,b. Its effectiveness in enhancing HE resistance is further promoted by the graded distribution of Mn [75, 76]. The Mn content varies continuously from approximately 0 at% in the Fe_50_Ni‐rich region to approximately 20 at% in the CoCrFeMnNi‐rich region, as shown in Figure 2b and Figure S13. Specifically, Mn‐rich segregation zones on the CoCrFeMnNi‐rich side (Figure 2c) increase plastic deformation capability, hydrogen trap density, and effectively reduce hydrogen diffusivity [77], thereby passivating and arresting the propagation of hydrogen‐induced microcracks [78, 79, 80]. In fact, the hydrogen diffusion coefficient of CoCrFeMnNi is approximately 1.75 × 10^−^^1^ ^1^ m^2^/s [81], while that of Fe_50_Ni is about 1.5 × 10^−^^1^ ^2^ m^2^/s [82], indicating that the value for the former is one order of magnitude higher than that for the latter. However, it should be noted that a higher diffusion coefficient merely reflects faster kinetic transport; the local hydrogen concentration is also governed by solubility, trap density, trapping binding energy, and the chemical potential. As supported by the MD simulation in Figure 10c, the CoCrFeMnNi‐rich region is more prone to generate nanotwins. Hydrogen sufficiently stimulates the generation of long‐range SFs and slips by lowering the SFE [83, 84], promoting the nucleation of nanotwins during plastic deformation [85]. This interpretation is corroborated by finite element simulations of hydrogen distribution across the compositional gradient (see Figures S14 and S15), which indicate that both hydrogen enrichment and diffusion depth are significantly more pronounced in the CoCrFeMnNi‐rich layers than in the Fe_50_Ni‐rich layers. The gradient nanotwin structure generated within the low‐SFE matrix in the CoCrFeMnNi‐rich region during deformation confers robust HE resistance, thereby synergistically enhancing the overall hydrogen compatibility of the graded alloy. It should be acknowledged, however, that direct spatially resolved hydrogen quantification at the nanoscale, particularly concerning the interaction between deformation twins and hydrogen, has not been achieved in the present work. This limitation points to a critical avenue for future research, where advanced characterization tools such as cryogenic atom probe tomography or nano‐scale secondary ion mass spectrometry could provide direct experimental evidence.

Consequently, a graded alloy that synergistically integrates the high SFE and low hydrogen diffusivity of the Fe_50_Ni‐rich region with the low SFE of the CoCrFeMnNi‐rich region is expected to yield superior hydrogen compatibility. Moreover, TEM analysis in Figure 11c,d of this region confirms the absence of mechanically load‐driven martensite transformation, which avoids the occurrence of HE that could otherwise be induced by potential phase boundaries acting as reversible hydrogen traps [86].

TEM observations in Figure 11c,d also reveal the absence of cellular structures. The additively manufacturing‐induced dislocation cellular structures become dissociation during the hydrogen‐charging process [87], correlating with the observed YS drop compared to the uncharged sample at 298 K (Figure 3a). The decrease in strength is ascribed to the corresponding weakening of the cellular structure's pinning effect on dislocation motion [87, 88]. To accommodate deformation in the CoCrFeMnNi‐rich region (governed by dislocation‐nanotwin interactions), the Fe_50_Ni‐rich region develops geometrically necessary boundaries (GNBs), incidental dislocation boundaries (IDBs), and dislocation walls (DWs). Therefore, the deformed microstructure exhibits significant lattice distortion, as shown in the Fe_50_Ni‐rich region and its corresponding GPA in Figure S11. As shown Figure 11e, the material exhibits a total hydrogen content of 2.64 ppmw. The deconvoluted peaks 1, 2, and 3 correspond to hydrogen trapped at lattice sites, grain boundaries/dislocation cores, and vacancies, respectively [89, 90]. Comparison of the peak area fractions reveals that Peak 1 (lattice sites) exhibits the highest intensity, accounting for 81.3% of the total, while Peaks 2 (grain boundaries/dislocation cores) and 3 (vacancies) show similar intensities. This suggests that hydrogen is primarily stored as interstitial atoms within the lattice, thereby inducing lattice distortion. Therefore, HR‐TEM imaging of the CoCrFeMnNi‐rich region (Figure 11f) reveals a distorted atomic plane with an interplanar spacing of approximately 0.104 nm on the (111) plane.

Additionally, nanotwins and SFs are visible in Figure 11g1, and their presence is confirmed by the SAED pattern in Figure 11g2. These coherent twin boundaries, in turn, act as barriers to hydrogen diffusion in the lattice during plastic deformation [19, 91]. Consequently, the sample effectively delivers desired performance in hydrogen‐exposed environments, addressing the challenge of integrating deformation mechanisms across a compositional gradient.

## 5 Conclusions

In this study, we systematically investigated the mechanical properties of an additively manufactured gradient (CoCrMn)_100‐2x_Fe_x_Ni_x_ alloy for extreme service environments, specifically examining its mechanical behavior at cryogenic temperatures and the influence of internal hydrogen charging on its tensile deformation response at 298 K. The main findings are summarized as follows:

1. Through in situ alloying via direct energy deposition combined with thermodynamic phase calculations, the composition of Fe_50_Ni and CoCrFeMnNi HEA was precisely tailored to fabricate a gradient (CoCrMn)_100‐2x_Fe_x_Ni_x_ alloy. A single FCC phase and dislocation cells were observed throughout the build.
2. The gradient specimen exhibits synergistically optimized mechanical properties at cryogenic temperatures, demonstrating significantly enhanced strength with decreasing temperature. DPF is substantially suppressed at ∼20 K, as evidenced by minor stress drops (<1.5 MPa), compared with ∼20 MPa in the CoCrFeMnNi counterpart.
3. The elimination of DPF is attributed to enhanced cryogenic deformation compatibility, which promotes the activation of multiple dislocation slip systems and suppresses excessive planar slip. Comprehensive EBSD‐TEM characterizations, combined with DFT calculations and MD simulations, reveal that the CoCrFeMnNi‐rich region, characterized by its low intrinsic SFE, facilitates the formation of stacking faults and deformation twins, particularly at cryogenic temperatures. These defects act as effective barriers to dislocation motion, thereby mediating planar slip. In contrast, the Fe_50_Ni‐rich layer, owing to its high SFE, does not develop deformation twins; even at 20 K, only sharpened dislocation cells and deformation bands are observed. Nevertheless, this layer effectively accommodates dislocation slip pathways, contributing to overall plastic compatibility.
4. The elimination of HE at 298 K is due to the synergistic interaction between the chemical gradient, particularly of Mn, and the gradient twin distributions, tailored through SFE control during plastic deformation.

This work establishes a new paradigm for designing and fabricating structural‐functional alloys, mechanistically validating that a continuous SFE gradient architecture can independently suppress cryogenic DPF (20 K, inert) and maintain HE resistance (298 K, charged) under separate assessments. However, the coupled cryogenic hydrogen condition has not been experimentally validated here and remains a key future priority. Nevertheless, the present decoupled approach systematically deconvolutes the individual contributions of the gradient architecture to each degradation mode, thereby establishing a foundation for rational alloy design in future hydrogen applications. Future efforts may also focus on enhancing overall mechanical performance through the deliberate introduction of additional beneficial lattice defects, such as trace interstitial atoms (B, N etc.), dislocations, precipitates, and nanotwins, to further optimize mechanical performance [88].

## Author Contributions

**Renhao Wu**: Project administration, Conceptualization, Methodology, Data curation, Validation, Writing – original draft, review & editing. **Ao Geng**: Investigation, Data curation, Methodology, Visualization, Validation. **Peihao Geng**: Project administration, Supervision, Methodology, Investigation, Funding acquisition, Writing – review& editing. **Hyojin Park**: Data curation, Investigation, Methodology. **Eun Seong Kim**: Investigation, Methodology. **Sang‐Ho Oh**: Data curation, Methodology. **Stephan Schönecker**: Data curation, Methodology. **Xiaoqing Li**: Data curation, Methodology, Supervision. **Shuhui Li**: Investigation, Resources, Methodology. **Tianlong Zhang**: Investigation, Supervision, Methodology, Writing – review & editing. **Byeong‐Joo Lee**: Investigation, Supervision, Methodology. **Hyoung Seop Kim**: Supervision, Funding acquisition, Project administration, Resources, Writing – review & editing.

## Conflicts of Interest

The authors declare no conflicts of interest.

## Supporting information




**Supporting File 1**: advs77732‐sup‐0001‐SuppMat.zip.

**Supporting File 2**: advs77732‐sup‐0002‐SuppMat.docx.

## Data Availability

The data that support the findings of this study are available on request from the corresponding author. The data are not publicly available due to privacy or ethical restrictions.

## References

[1] A. Tehranchi , X. Zhou , and W. A. Curtin , “A Decohesion Pathway for Hydrogen Embrittlement in Nickel: Mechanism and Quantitative Prediction,” Acta Materialia 185 (2020): 98–109.

[2] B. Gludovatz , A. Hohenwarter , D. Catoor , E. H. Chang , E. P. George , and R. O. Ritchie , “A Fracture‐Resistant High‐Entropyalloy for Cryogenic Applications,” Science 345, no. 6201 (2014): 1153–1158. 25190791 10.1126/science.1254581

[3] T. Chen , H. Su , M. Koyama , et al., “Hydrogen Embrittlement in a CoCrNi Medium‐Entropy Alloy Fabricated via Laser Powder Bed Fusion: Characteristic Intergranular Cracking and Hydrogen‐Enhanced Twinning,” Journal of Materials Science & Technology 240 (2026): 201–213.

[4] T. Lu , B. Sun , Y. Li , et al., “Dual‐Scale Chemical Ordering for Cryogenic Properties in CoNiV‐Based Alloys,” Nature 645, no. 8080 (2025): 385–391. 40866698 10.1038/s41586-025-09458-1 PMC12422977

[5] J. Tabin , B. Skoczeń , and J. Bielski , “Discontinuous Plastic Flow in Stainless Steels Subjected to Combined Loads at Extremely Low Temperatures,” International Journal of Mechanical Sciences 200 (2021): 106448.

[6] Y. S. Kim , T. Kang , S.‐K. Hong , et al., “Fundamental Mechanisms of Discontinuous Deformation in Metals for Cryogenic‐Environment Applications,” Acta Materialia 292 (2025): 120970.

[7] X.‐Y. Zhou , H.‐H. Wu , M. Zhou , L. Wang , T. Lookman , and X. Mao , “Enhanced Hydrogen Embrittlement Resistance of FeCoNiCrMn Multi‐Principal Element Alloys via Local Chemical Ordering and Grain Boundary Segregation,” Acta Materialia 296 (2025): 121209.

[8] H. Yu , A. Díaz , X. Lu , et al., “Hydrogen Embrittlement as a Conspicuous Material Challenge─Comprehensive Review and Future Directions,” Chemical Reviews 124, no. 10 (2024): 6271. 38773953 10.1021/acs.chemrev.3c00624 PMC11117190

[9] J. H. Liang , J. L. Sun , S. J. Qu , et al., “Additively‐Manufactured High‐Entropy Cantor Alloy: Multiscale Microstructures and Deformation Mechanisms,” Materials Science and Engineering: A 946 (2025): 149096.

[10] T.‐H. Lee , E. Shin , C.‐S. Oh , H.‐Y. Ha , and S.‐J. Kim , “Correlation Between Stacking Fault Energy and Deformation Microstructure in High‐Interstitial‐Alloyed Austenitic Steels,” Acta Materialia 58, no. 8 (2010): 3173–3186.

[11] M. Beyramali Kivy and M. A. Zaeem , “Generalized Stacking Fault Energies, Ductilities, and Twinnabilities of CoCrFeNi‐Based Face‐Centered Cubic High Entropy Alloys,” Scripta Materialia 139 (2017): 83–86.

[12] D. Lin , J. Hu , R. Wu , et al., “Multiscale Plastic Deformation in Additively Manufactured FeCoCrNiMo High‐Entropy Alloys to Achieve Strength–Ductility Synergy at Elevated Temperatures,” International Journal of Plasticity 183 (2024): 104142.

[13] P. Niu , R. Li , K. Gan , Z. Fan , T. Yuan , and C. Han , “Manipulating Stacking Fault Energy to Achieve Crack Inhibition and Superior Strength‐Ductility Synergy in an Additively Manufactured High‐Entropy Alloy,” Advanced Materials 36 (2024): 2310160. 10.1002/adma.202310160 38489830

[14] X. Li , S. Schönecker , L. Vitos , and X. Li , “Generalized Stacking Faults Energies of Face‐Centered Cubic High‐Entropy Alloys: A First‐Principles Study,” Intermetallics 145 (2022): 107556.

[15] Z. Dong , W. Li , G. Chai , and L. Vitos , “Strong Temperature—Dependence of Ni ‐Alloying Influence on the Stacking Fault Energy in Austenitic Stainless STEEL,” Scripta Materialia 178 (2020): 438–441.

[16] J. Yang , X. Su , Y. Li , Z. Zhang , J. Zhou , and X. Zhang , “Origin of the Discontinuous Plastic Flow and Anomalous Elongation‐to‐Failure for 316LN Stainless Steel Under Ultra‐Low Temperatures,” Materials Science and Engineering: A 923 (2025): 147670.

[17] H.‐J. Kim , M.‐K. Cho , G. Kim , et al., “Influence of Hydrogen Absorption on Stacking Fault of Energy of a Face‐Centered Cubic High Entropy Alloy,” Metals and Materials International 28, no. 11 (2022): 2637–2645.

[18] Y. Zhu , Z. Zheng , M. Huang , S. Liang , and Z. Li , “Modeling of Solute Hydrogen Effect on Various Planar Fault Energies,” International Journal of Hydrogen Energy 45, no. 15 (2020): 9162–9173.

[19] H. Luo , W. Lu , X. Fang , D. Ponge , Z. Li , and D. Raabe , “Beating Hydrogen With Its Own Weapon: Nano‐Twin Gradients Enhance Embrittlement Resistance of a High‐Entropy Alloy,” Materials Today 21, no. 10 (2018): 1003–1009.

[20] B. Sun , W. Lu , B. Gault , et al., “Chemical Heterogeneity Enhances Hydrogen Resistance in High‐Strength Steels,” Nature Materials 20, no. 12 (2021): 1629–1634. 34239084 10.1038/s41563-021-01050-y PMC8610813

[21] N. Sargent , S. Firdosy , X. Wang , et al., “Discovery of Microsegregation‐Aided Transformation and Twinning‐Induced Plasticity in Low Mn Steel Through Directed Energy Deposition of Functionally Graded Materials,” Additive Manufacturing 85 (2024): 104154.

[22] T. Z. Khan , T. Kirk , G. Vazquez , et al., “Towards Stacking Fault Energy Engineering in FCC High Entropy Alloys,” Acta Materialia 224 (2022): 117472.

[23] P. Sathiyamoorthi and H. S. Kim , “High‐Entropy Alloys With Heterogeneous Microstructure: Processing and Mechanical Properties,” Progress in Materials Science 123 (2022): 100709.

[24] J. Liu , X. Guo , Q. Lin , et al., “Excellent Ductility and Serration Feature of Metastable CoCrFeNi High‐Entropy Alloy at Extremely Low Temperatures,” Science China Materials 62, no. 6 (2018): 853–863.

[25] D. Gu , X. Shi , R. Poprawe , D. L. Bourell , R. Setchi , and J. Zhu , “Material‐Structure‐Performance Integrated Laser‐Metal Additive Manufacturing,” Science 372, no. 6545 (2021): eabg1487. 34045326 10.1126/science.abg1487

[26] P. Hohenberg and W. Kohn , “Inhomogeneous Electron Gas,” Physical review 136, no. 3B (1964): B864–B871.

[27] L. Vitos , “Total‐Energy Method Based on the Exact Muffin‐Tin Orbitals Theory,” Physical Review B 64, no. 1 (2001): 014107.

[28] J. P. Perdew , K. Burke , and M. Ernzerhof , “Generalized Gradient Approximation Made Simple,” Physical review letters 77, no. 18 (1996): 3865–3868. 10062328 10.1103/PhysRevLett.77.3865

[29] B. Gyorffy , A. Pindor , J. Staunton , G. Stocks , and H. Winter , “A First‐Principles Theory of Ferromagnetic Phase Transitions in Metals,” Journal of Physics F: Metal Physics 15, no. 6 (1985): 1337–1386.

[30] L. Vitos , I. A. Abrikosov , and B. Johansson , “Anisotropic Lattice Distortions in Random Alloys From First‐Principles Theory,” Physical Review Letters 87, no. 15 (2001): 156401. 11580714 10.1103/PhysRevLett.87.156401

[31] F. Körmann , D. Ma , D. D. Belyea , et al., “Treasure Maps″ for Magnetic High‐Entropy‐Alloys From Theory and Experiment,” Applied Physics Letters 107, no. 14 (2015): 142404.

[32] W.‐M. Choi , Y. H. Jo , S. S. Sohn , S. Lee , and B.‐J. Lee , “Understanding the Physical Metallurgy of the CoCrFeMnNi High‐Entropy Alloy: An Atomistic Simulation Study,” npj Computational Materials 4, no. 1 (2018): 1.

[33] J. B. Seol , W.‐S. Ko , S. S. Sohn , et al., “Mechanically Derived Short‐Range Order and Its Impact on the Multi‐Principal‐Element Alloys,” Nature Communications 13, no. 1 (2022): 6766. 10.1038/s41467-022-34470-8 PMC9646780 36351925

[34] Q. Zhou , Z. Jiao , Z. Huang , et al., “Wear‐Resistant CrCoNi Nanocrystalline Film via Friction‐Driven Surface Segregation,” Acta Materialia 279 (2024): 120299.

[35] W. G. Hoover , “Canonical Dynamics: Equilibrium Phase‐Space Distributions,” Physical Review A 31, no. 3 (1985): 1695–1697. 10.1103/physreva.31.1695 9895674

[36] W. G. Hoover , “Constant‐Pressure Equations of Motion,” Physical Review A 34, no. 3 (1986): 2499–2500. 10.1103/physreva.34.2499 9897546

[37] X. Li , Y. Wei , L. Lu , K. Lu , and H. Gao , “Dislocation Nucleation Governed Softening and Maximum Strength in Nano‐Twinned Metals,” Nature 464, no. 7290 (2010): 877–880. 20376146 10.1038/nature08929

[38] Z. Cheng , H. Zhou , Q. Lu , H. Gao , and L. Lu , “Extra Strengthening and Work Hardening in Gradient Nanotwinned Metals,” Science 362, no. 6414 (2018): aau1925. 10.1126/science.aau1925 30385547

[39] R. Wu , H. Park , J. H. Lee , et al., “Engineering Stacking Fault Energy and Hierarchical Precipitates in a Near‐Fully Recrystallized DED Ni‐Based Multi‐Principal Element Alloy,” International Journal of Plasticity 201 (2026): 104682.

[40] H.‐S. Do , W.‐M. Choi , and B.‐J. Lee , “A Thermodynamic Description for the Co–Cr–Fe–Mn–Ni system,” Journal of Materials Science 57, no. 2 (2022): 1373–1389.

[41] C. Körner , M. Markl , and J. A. Koepf , “Modeling and Simulation of Microstructure Evolution for Additive Manufacturing of Metals: A Critical Review,” Metallurgical and Materials Transactions A 51, no. 10 (2020): 4970–4983.

[42] Y. Zhou , X. Li , C. Guo , X. Hu , and Q. Zhu , “Investigation of Columnar to Equiaxial Transition Criterion and Solidification Conditions for Ni‐Based Superalloy in Laser Powder Bed Fusion,” Journal of Alloys and Compounds 966 (2023): 171611.

[43] R. Brooke , D. Zhang , D. Qiu , M. A. Gibson , and M. Easton , “Compositional Criteria to Predict Columnar to Equiaxed Transitions in Metal Additive Manufacturing,” Nature Communications 16, no. 1 (2025): 5710. 10.1038/s41467-025-60162-0 PMC12216522 40593505

[44] A. S. Tirunilai , T. Hanemann , K. P. Weiss , J. Freudenberger , M. Heilmaier , and A. Kauffmann , “Dislocation‐Based Serrated Plastic Flow of High Entropy Alloys at Cryogenic Temperatures,” Acta Materialia 200 (2020): 980–991.

[45] W. Kucza , J. Dąbrowa , G. Cieślak , K. Berent , T. Kulik , and M. Danielewski , “Studies of “Sluggish Diffusion” effect in Co‐Cr‐Fe‐Mn‐Ni, Co‐Cr‐Fe‐Ni and Co‐Fe‐Mn‐Ni High Entropy Alloys; Determination of Tracer Diffusivities by Combinatorial Approach,” Journal of Alloys and Compounds 731 (2018): 920–928.

[46] C. Bulut , F. Yıldız , T. Varol , G. Kaya , and O. E. Tevfik , “Effects of Selective Laser Melting Process Parameters on Structural, Mechanical, Tribological and Corrosion Properties of CoCrFeMnNi High Entropy Alloy,” Metals and Materials International 30, no. 11 (2024): 2982–3004.

[47] B. Wang , J. Liu , Z. Qin , et al., “Cumulative Strain‐Induced Gradient Heterostructure for Synchronously Boosting Mechanical Properties and Corrosion Resistance,” Journal of Materials Science & Technology 270 (2026): 1–14.

[48] H. Shahmir , M. S. Mehranpour , S. A. Arsalan Shams , and T. G. Langdon , “Twenty Years of the CoCrFeNiMn High‐Entropy Alloy: Achieving Exceptional Mechanical Properties Through Microstructure Engineering,” Journal of Materials Research and Technology 23 (2023): 3362–3423.

[49] T. K. Liu , Z. Wu , A. D. Stoica , et al., “Twinning‐Mediated Work Hardening and Texture Evolution in CrCoFeMnNi High Entropy Alloys at Cryogenic Temperature,” Materials & Design 131 (2017): 419–427.

[50] Z. S. Toor , R. Wu , M. R. Hashmi , et al., “Computation‐ and Process‐Based Design for Advanced Structural High‐Entropy Alloy Development and Analyses: A Critical Review,” Progress in Materials Science 155 (2026): 101534.

[51] K. Lu , “Stabilizing Nanostructures in Metals Using Grain and Twin Boundary Architectures,” Nature Reviews Materials 1, no. 5 (2016): 16019.

[52] K. Nalepka , J. Tabin , J. Kawałko , A. Brodecki , P. Bała , and Z. Kowalewski , “Plastic Flow Instability in Austenitic Stainless Steels at Room Temperature: Macroscopic Tests and Microstructural Analysis,” International Journal of Plasticity 183 (2024): 104159.

[53] D. Utt , S. Lee , Y. Xing , et al., “The Origin of Jerky Dislocation Motion in High‐Entropy Alloys,” Nature Communications 13, no. 1 (2022): 4777. 10.1038/s41467-022-32134-1 PMC9378647 35970838

[54] Q.‐J. Li , H. Sheng , and E. Ma , “Strengthening in Multi‐Principal Element Alloys With Local‐Chemical‐Order Roughened Dislocation Pathways,” Nature Communications 10, no. 1 (2019): 3563. 10.1038/s41467-019-11464-7 PMC6687833 31395881

[55] J. Tabin , B. Skoczen , and J. Bielski , “Strain Localization During Discontinuous Plastic Flow at Extremely Low Temperatures,” International Journal of Solids and Structures 97‐98 (2016): 593–612.

[56] T. J. Jang , G. Lee , S. Y. Song , et al., “Ultrastrong and Ductile High‐Entropy Alloy With Minimal Serration at Liquid Helium Temperature via Coherent Nanoprecipitate,” Acta Materialia 300 (2025): 121513.

[57] A. S. Tirunilai , T. Hanemann , C. Reinhart , et al., “Comparison of Cryogenic Deformation of the Concentrated Solid Solutions CoCrFeMnNi, CoCrNi and CoNi,” Materials Science and Engineering: A 783 (2020): 139290.

[58] J. Moon , E. Tabachnikova , S. Shumilin , et al., “Deformation Behavior of a Co‐Cr‐Fe‐Ni‐Mo Medium‐Entropy Alloy at Extremely Low Temperatures,” Materials Today 50 (2021): 55–68.

[59] Y. Zhang , W. Yang , J. Peng , A. Wang , W. Fan , and J. Li , “Unveiling Wear Property and Multiscale Tribological Mechanism of Laser Cladding‐Nitriding Synergistically Enhanced High‐Entropy Alloy Coatings,” Applied Surface Science 710 (2025): 163878.

[60] T. Chang , M. Ma , H. Zhang , X. Fang , and K. Huang , “Synergistic Performance Enhancement of Additively Manufactured Copper‐Nickel Dissimilar Alloy Components via a Novel Quasi in Situ Cryogenic Treatment,” International Journal of Extreme Manufacturing 8, no. 4 (2026): 045003.

[61] Y. Wei , Y. Li , L. Zhu , et al., “Evading the Strength–Ductility Trade‐Off Dilemma in Steel through Gradient Hierarchical Nanotwins,” Nature Communications 5, no. 1 (2014): 3580. 10.1038/ncomms4580 PMC3988817 24686581

[62] G. Potnis , D. Goswami , and J. Das , “Twinning Mediated Plasticity in High Entropy CoCr_1.3_FeNi_0.7_MnNb (x = 0.3, 0.367, 0.4) Ultrafine Lamellar Eutectic by Tuning Stacking Fault Energy,” Scripta Materialia 227 (2023): 115271.

[63] D. W. Lee , J. A. Lee , R. Wu , et al., “Tailoring co‐Sintering Mechanism and Mechanical Properties in STS316L‐IN718 Layered Composite via Dual‐Nozzle Material Extrusion,” Materials Science and Engineering: A 969 (2026): 150460.

[64] M. Jin , B. Lee , J. Yoo , Y. Jo , and S. Lee , “Cryogenic Deformation Behaviour of Aluminium Alloy 6061‐T6,” Metals and Materials International 30, no. 6 (2024): 1492–1504.

[65] A. K. Chandan , P. Murugaiyan , and S. G. Chowdhury , “Temperature‐Dependent Stacking Fault Energy, Deformation Behavior, and Tensile Properties of a New High‐Entropy Alloy,” Materials Science and Engineering: A 883 (2023): 145522.

[66] E. Ma and X. Wu , “Tailoring Heterogeneities in High‐Entropy Alloys to Promote Strength–Ductility Synergy,” Nature Communications 10, no. 1 (2019): 5623. 10.1038/s41467-019-13311-1 PMC6901531 31819051

[67] F. Otto , A. Dlouhý , C. Somsen , H. Bei , G. Eggeler , and E. P. George , “The Influences of Temperature and Microstructure on the Tensile Properties of a CoCrFeMnNi High‐Entropy Alloy,” Acta Materialia 61, no. 15 (2013): 5743–5755.

[68] B. Xia and H. Wang , “Evaluation of Cryogenic Toughness Variation in Multi‐Pass HAZ of High Mn Austenitic Steel for LNG Cryogenic Application,” Metals and Materials International 32, no. 3 (2025): 946–955.

[69] G. Laplanche , A. Kostka , O. M. Horst , G. Eggeler , and E. P. George , “Microstructure Evolution and Critical Stress for Twinning in the CrMnFeCoNi High‐Entropy Alloy,” Acta Materialia 118 (2016): 152–163.

[70] J. He , F. Yuan , M. Yang , L. Zhou , S. Jiao , and X. Wu , “Exceptional Tensile Properties Under Cryogenic Temperature in Heterogeneous Laminates Induced by Non‐Uniform Martensite Transformation and Strain Delocalization,” Materials Science and Engineering: A 791 (2020): 139780.

[71] Y. Zhang , Z. Chen , Z. Liu , H. Wu , and G. Fan , “Layer‐Thickness‐Dependent Unusual Strain Hardening of Ti/Ti_6_Al_4_V Laminated Composites: An in Situ Cryogenic EBSD Study,” Journal of Materials Science & Technology 259 (2026): 322–336.

[72] E. Antillon , C. Woodward , S. I. Rao , B. Akdim , and T. A. Parthasarathy , “A Molecular Dynamics Technique for Determining Energy Landscapes as a Dislocation Percolates Through a Field of Solutes,” Acta Materialia 166 (2019): 658–676.

[73] Y. T. Zhu , J. Narayan , J. P. Hirth , S. Mahajan , X. L. Wu , and X. Z. Liao , “Formation of Single and Multiple Deformation Twins in Nanocrystalline Fcc Metals,” Acta Materialia 57, no. 13 (2009): 3763–3770.

[74] J. W. Christian and S. Mahajan , “Deformation Twinning,” Progress in Materials Science 39, no. 1‐2 (1995): 1–157.

[75] Y. Xu , H. Toda , K. Shimizu , et al., “Suppressed Hydrogen Embrittlement of High‐Strength Al Alloys by Mn‐Rich Intermetallic Compound Particles,” Acta Materialia 236 (2022): 118110.

[76] X. Li , Z. Feng , X. Song , Y. Wang , and Y. Zhang , “Effect of Hydrogen Charging Time on Hydrogen Embrittlement of CoCrFeMnNi High‐Entropy Alloy,” Corrosion Science 198 (2022): 110073.

[77] S. Jamal , Y. Wang , M. M. Abu Bakar Baig , et al., “Deciphering the Role of Manganese‐Alloying on Hydrogen Embrittlement Susceptibility of Martensitic Steel,” Corrosion Science 263 (2026): 113728.

[78] J. H. Ryu , Y. S. Chun , C. S. Lee , H. K. D. H. Bhadeshia , and D. W. Suh , “Effect of Deformation on Hydrogen Trapping and Effusion in TRIP‐Assisted Steel,” Acta Materialia 60, no. 10 (2012): 4085–4092.

[79] D. Raabe , C. C. Tasan , and E. A. Olivetti , “Strategies for Improving the Sustainability of Structural Metals,” Nature 575, no. 7781 (2019): 64–74. 31695209 10.1038/s41586-019-1702-5

[80] T. M. Park , H.‐J. Kim , H. Y. Um , N. H. Goo , and J. Han , “The Possibility of Enhanced Hydrogen Embrittlement Resistance of Medium‐Mn Steels by Addition of Micro‐Alloying Elements,” Materials Characterization 165 (2020): 110386.

[81] J. Lee , C. Park , H. Park , and N. Kang , “Effective Hydrogen Diffusion Coefficient for CoCrFeMnNi High‐Entropy Alloy and Microstructural Behaviors After Hydrogen Permeation,” International Journal of Hydrogen Energy 45, no. 16 (2020): 10227–10232.

[82] T. Otsuka , S. Sasabe , and T. Tanabe , “Hydrogen diffusion in Fe–Ni alloys Around room temperature,” Journal of Nuclear Materials 386‐388 (2009): 884–887.

[83] A. E. Pontini and J. D. Hermida , “X‐Ray Diffraction Measurement of the Stacking Fault Energy Reduction Induced by Hydrogen in an AISI _304_ Steel,” Scripta Materialia 37, no. 11 (1997): 1831–1837.

[84] D. Feng , J. Xu , Y. Zheng , et al., “High‐Entropy Alloy near‐Surface Hydrogen‐Induced Nanotwin and Spinodal Decomposition Promoting Heterogeneous Structure Evolution,” Materials Research Letters 14, no. 1 (2026): 20–28.

[85] X. Li , H. Zhang , J. Jia , J. Liu , and Y. Zhang , “A Twinning Mechanism: Direct Observation of the Transition From Stacking Faults to Microtwins,” Scripta Materialia 267 (2025): 116803.

[86] R. Wu , Z. S. Toor , M. R. Hashmi , et al., “Multiscale Computational Design of Hydrogen‐Compatible Integrated Structural‐Functional Alloys,” Progress in Materials Science 162 (2026): 101727.

[87] S.‐H. Li , D.‐H. Lee , Y. Zhao , and U. Ramamurty , “Hydrogen‐Induced Softening and Embrittlement in 316L Stainless Steel Fabricated Using Laser‐Powder Bed Fusion,” Acta Materialia 274 (2024): 119959.

[88] H. Cheng , H. Luo , Z. Pan , et al., “Effects of Laser Powder Bed Fusion Process Parameters on Microstructure and Hydrogen Embrittlement of High‐Entropy Alloy,” Journal of Materials Science & Technology 155 (2023): 211–226.

[89] Z. Gao , D.‐H. Lee , Y. Zhao , et al., “Hydrogen Trapping and Micromechanical Behavior in Additively Manufactured CoCrFeNi High‐Entropy Alloy in as‐Built and Pre‐Strained Conditions,” Acta Materialia 271 (2024): 119886.

[90] J. Kangazian , S. Y. Ahn , B. W. Koo , et al., “Hydrogen Embrittlement Mitigation via Microstructural and Phase Control in Laser Powder bed–Fused SAF 3207 Hyper Duplex Stainless Steel,” Materials Science and Engineering: A 955 (2026): 149861.

[91] R. Wu , S. Y. Ahn , Y. T. Choi , et al., “Fostering Strengths Against Hydrogen Embrittlement: Insights From Nanotwin‐Ability and Post‐Treatment Effects in Additively Manufactured CoCrFeMnNi,” Materials Research Letters 12, no. 10 (2024): 689–699.

