# Supplementary material for: A 3D‐Printed SFE‐Graded Alloy Mitigates Cryogenic Discontinuous Plastic Flow and Hydrogen Embrittlement
Source: Adv Sci (Weinh). 2026 Sep 11:e77732. Online ahead of print. doi: 10.1002/advs.77732 (PMC13564189; doi:10.1002/advs.77732)
Supplement: Supplementary file 1 [file ADVS-9999-e77732-s002.docx]

***Supplementary Materials***

**A 3D-printed SFE-graded alloy mitigates cryogenic discontinuous plastic flow and hydrogen embrittlement**

**Fig. S1.** Illustration of experimental design and DED process parameters.

**Fig. S2.** (a) Fabricated blocks by in situ alloying DED process and the dog-bone tensile specimen, microstructure characterizations in randomly selected specimen to show the fine print quality, grain and chemical gradient; (b) Size of dog-bone tensile specimen and locations for microstructural characterization.

In Fig. S3, the twinning nucleation stress revealed that Fe_50_Ni and CoCrFeMnNi exhibit a reduction in twinning nucleation stress with increasing temperature, whereas Co_10_Cr_10_Fe_35_Mn_10_Ni_35_ would not follow the same trend.

**Fig. S3.** MD simulation of the tensile stress-strain response of different single-component materials. (a) Simulation setup; (b) Fe_50_Ni, (c) Co_10_Cr_10_Fe_35_Mn_10_Ni_35_, (d) CoCrFeMnNi.

To gain deeper insight into the deformation mechanisms of layered compositions nanolaminate (Fe_50_Ni/Co_10_Cr_10_Fe_35_Mn_10_Ni_35_/CoCrFeMnNi), we also constructed a tensile simulation on the nanocrystalline model (Fig. S4a) containing 64 grains. The nanoscale stress-strain responses under uniaxial tension (Fig. S4b) demonstrate that 20 K significantly suppresses dislocation activity, thereby elevating the critical yield condition. Figure S4c displays dislocation densities in Fe_50_Ni, Co_10_Cr_10_Fe_35_Mn_10_Ni_35_, and CoCrFeMnNi during tensile deformation at 20 K and 298 K.

**Fig. S4.** MD simulations of uniaxial tensile deformation in nanolaminates with different compositions. (a) Schematic representation of the simulated nanolaminate grain structures, where periodic boundary conditions generate repeated grains, for three model alloys: No.1 (Fe_50_Ni), No.2 (Co_10_Cr_10_Fe_35_Mn_10_Ni_35_), and No.3 (CoCrFeMnNi). (b-c) Stress-strain responses and corresponding dislocation density evolution at 20 K and 298 K.

**Fig. S5.** EBSD characterization of the as-DED-processed gradient (CoCrMn)_100-2x_Fe_x_Ni_x_ sample: (a) detailed recrystallization status with (b) local GND distribution at the critical CET area in the as-fabricated sample.

**Fig. S6.** TEM characterization coupled with EDS elemental mapping of Fe_50_Ni-rich side in the as-fabricated sample.

**Fig. S7.** Spatial variation of elemental composition in the as-fabricated sample. EDS point scans quantify the atomic ratios across the Fe_50_Ni-rich and CoCrFeMnNi-rich sides.

**Table S1** Mechanical properties of gradient sample under different conditions at tensile strain of 1 × 10^-4^ s^-1^.

| **Condition** | **YS, MPa** | **UTS, MPa** | **UFE** | **TE** |
| --- | --- | --- | --- | --- |
| 20K | 613.6 | 891.1 | 0.481 | 0.502 |
| 77K | 591.1 | 824.7 | 0.363 | 0.432 |
| 150K | 472.9 | 643.2 | 0.242 | 0.372 |
| 298K | 355.7 | 497.86 | 0.1988 | 0.3956 |
| 298K-H | 323.1 | 460.3 | 19.88 | 0.394 |

**Fig. S8.** Partially magnified stress-strain curves to observe suppressed discontinuous plastic flow. (a) 77K, (b) 150K, (c) 298K, (d) 298K - H charged.

**Fig. S9.** (a) Calculated solidification path for the gradient alloy using the Scheil-Gulliver model, simulating DED non-equilibrium rapid cooling. (b) Mole fraction of all elements vs. solid fraction in the in-situ alloying layer.

**Fig. S10.** Gradient (CoCrMn)_100-2x_Fe_x_Ni_x_ sample deformed at 298K. (a) Cross-sectional ECCI/IPF overview of the near-fracture region, linking the Fe₅₀Ni-rich and CoCrFeMnNi-rich sides. (b) local Fe_50_Ni-rich region: (b1) band contrast and (b2) GND map (avg. 7.91 × 10¹⁴ m⁻²) showing cross-grain slip traces. (c) local CoCrFeMnNi-rich region: (c1) band contrast and (c2) GND map (avg. 7.91 × 10¹⁴ m⁻²) revealing intra-granular slips and nanotwins.

**Fig. S11.** Stacking faults (SFs) in CoCrFeMnNi-rich region after 298K tensile deformation with Fast Fourier Transform (FFT) patterns.

**Table S2** DFT Calculated intrinsic SFE (mJ m^-2^) of typical compositions at FM/PM cases with deduced Curie temperature (Tc).

| **Nominal composition** | **FM case** | **PM case** | **Deduced Tc, K** |
| --- | --- | --- | --- |
| CoCrFeMnNi | -56.23 | -72.00 | 17.38 |
| Co_18_Cr_18_Fe_23_Mn_18_Ni_23_ | -29.03 | -30.00 | 45.26 |
| Co_16_Cr_16_Fe_26_Mn_16_Ni_26_ | -8.15 | -10.49 | 84.94 |
| Co_14_Cr_14_Fe_29_Mn_14_Ni_29_ | 10.81 | 8.74 | 152.73 |
| Co_10_Cr_10_Fe_35_Mn_10_Ni_35_ | 35.64 | 35.63 | 274.20 |
| Co_6_Cr_6_Fe_41_Mn_6_Ni_41_ | 59.36 | 53.85 | 417.88 |
| Fe_50_Ni | 100.16 | 71.87 | 522.19 |

Note: The aqua blue colored regions indicate the magnetic state (FM or PM) of corresponding composition and the predicted intrinsic SFE, determined by comparing the estimated Tc with 298 K.

**Fig. S12.** Scanning TEM characterization coupled with EDS elemental mapping of near Co_10_Cr_10_Fe_35_Mn_10_Ni_35_ region after tensile straining.

**Fig. S13.** The deformed microstructure shows significant lattice distortion in the Fe_50_Ni-rich region after hydrogen charging at 298 K and its corresponding GPA.

The effect of hydrogen diffusion driven by the concentration gradient across the gauge length was analyzed by employing finite element method. Commercially available ABAQUS software, using steady-state mass diffusion implicit step for 36 hours was utilized. The geometric model was designed having 6.4 mm × 1.2 mm dimensions of length and thickness. Eight different materials were assigned using partitioning based section assignment having the individual section thickness and diffusivities mentioned in Table S3. Diffusion coefficients for CoCrFeMnNi and Fe_50_Ni were sourced from Refs. [81, 82], with gradient‑layer values estimated by interpolation.

Table S3. Material section composition, thickness, and diffusion coefficient.

| **Composition** | **Thickness, mm** | **Hydrogen diffusion coefficient, m²/s** |
| --- | --- | --- |
| CoCrFeNiMn | 0.15 | 1.75 × 10^-11^ |
| CoCrFeNiMn | 0.15 | 1.75 × 10^-11^ |
| Co_18_Cr_18_Fe_23_Ni_23_Mn_18_ | 0.15 | 1.5 × 10^-11^ |
| Co_16_Cr_16_Fe_26_Mn_16_Ni_26_ | 0.15 | 1.05 × 10^-11^ |
| Co_14_Cr_14_Fe_29_Mn14Ni_29_ | 0.15 | 7.5 × 10^-12^ |
| Co_10_Cr_10_Fe_35_Mn_10_Ni_35_ | 0.15 | 4.5 × 10^-12^ |
| Co_6_Cr_6_Fe_41_Mn_6_Ni_41_ | 0.15 | 3 × 10^-12^ |
| Fe_50_Ni | 0.15 | 1.5 × 10^-12^ |

* The hydrogen diffusion coefficient of CoCrFeMnNi is one order of magnitude higher than that of Fe_50_Ni. However, it should be noted that a higher diffusion coefficient merely reflects faster kinetic transport; the local hydrogen concentration is also governed by solubility, trap density, trapping binding energy, and the chemical potential.

DC2D4 heat transfer elements were used for meshing of the model into77,561 nodes. The analysis emphasized hydrogen diffusion from both the top and bottom surfaces to support gradient-based simulations, as illustrated in Fig. S14.

**Fig. S14.** Assembled, meshed finite element model to calculate hydrogen concentration gradient in the thickness section of as-printed sample after diffusion for 36 hours.

The results of the transient diffusion of 36 hours hydrogen charging are compiled in Fig. S15. Hydrogen concentration maps revealed a prominent concentration gradient with different layered distributions from the top surface towards the bottom region, as shown in Fig. S15a. As highlighted in Fig. S15b, nodal assessment of the concentration along the thickness indicated a decreasing trend across the thickness till the center, followed by an increase till the bottom surface of the specimen. These results indicated that the hydrogen diffusion and concentration was sensitive to the gradient material distribution.

**Fig. S15.** Dual side diffusion simulation results in transient state after 36 hours. (a) Hydrogen concentration maps for 1.2 mm thickness and (b) Hydrogen concentration recorded in the thickness direction at nodal points.
